# Integrative Assessment of Hong Kong Chironomidae (Diptera) Shows High Species Richness Linked to Spatial and Environmental Factors

**DOI:** 10.1002/ece3.73110

**Published:** 2026-02-15

**Authors:** Wu Han, Tsz‐Ying Chan, Chu‐Ming Zhang, Xiao‐Long Lin, Peter S. Cranston, Thilina S. Nimalrathna, Bai‐An Lin, Hong‐Qu Tang, Mathew Seymour

**Affiliations:** ^1^ School of Biological Sciences The University of Hong Kong Hong Kong SAR China; ^2^ Engineering Research Center of Environmental DNA and Ecological Water Health Assessment Shanghai Ocean University Shanghai China; ^3^ Evolution & Ecology Australian National University Canberra Australian Capital Territory Australia; ^4^ Life Science and Technology College Jinan University Guangzhou China

**Keywords:** beta diversity, biogeography, Chironomidae, community assembly, DNA barcoding, Hong Kong

## Abstract

Inland waters face escalating anthropogenic pressures, driving an unprecedented collapse in freshwater biodiversity. Enhanced knowledge of aquatic taxa is essential to reverse this decline. Chironomidae (non‐biting midges), often the dominant zoobenthic group in freshwater ecosystems, remain poorly documented globally. Here, we provide the first integrative assessment of Chironomidae biodiversity in Hong Kong through a year‐long survey of five streams. Integrative taxonomy expanded the known species in Hong Kong from 17 to 243, and yielded a reference library of 827 cytochrome c oxidase subunit I (COI) barcodes representing 225 species. Beta‐diversity partitioning revealed that community dissimilarity was primarily driven by species turnover, which was strongly associated with environmental gradients but only weakly related to geographic distance. Variation partitioning revealed that environmental factors explained slightly more variation in community composition (9.0%) than spatial factors (6.7%). These patterns indicate that environmental filtering and mass effects play key roles in structuring Chironomidae metacommunities in Hong Kong, with dispersal limitation exerting little influence. Cross‐database barcode matching analysis suggested that Hong Kong fauna is predominantly tropical‐to‐subtropical, with the strongest affinities to coastal East and Southeast Asia (e.g., eastern China, Thailand, Malaysia). Many species displayed wide geographic ranges, likely facilitated by high passive dispersal and broad ecological tolerances. This study delivers the first robust biodiversity baseline for Hong Kong Chironomidae and a well‐curated DNA barcode library. These resources will benefit taxonomic refinement and eDNA‐based biomonitoring, strengthening conservation of human‐impacted freshwater ecosystems.

## Graphical abstract

This study presents the first comprehensive assessment of Chironomidae biodiversity in Hong Kong, expanding the known species count from 17 to 243 through integrative taxonomy and establishing a reference DNA barcode library of 827 COI sequences. Chironomidae metacommunities are shaped by environmental filtering and mass effects, with little evidence of dispersal limitation. At the global scale, Hong Kong's fauna shows strong biogeographic affinities to tropical and subtropical regions of East and Southeast Asia.

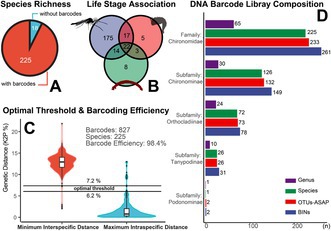

## 1 Introduction

Biodiversity underpins essential ecosystem functions and services crucial for human well‐being (Mace et al. 2012; Weiskopf et al. 2022). However, the Sixth Mass Extinction event has been triggered by human activities, manifesting in an unprecedented rate of species population decline and extinction (Cowie et al. 2022).

Biodiversity loss is particularly severe in freshwater systems, which support a disproportionately high share of global biodiversity despite covering only 0.8% of the Earth's surface (Strayer and Dudgeon 2010). Recent assessments reveal that biodiversity in roughly half of the world's rivers has been impacted, with particularly severe declines in densely populated catchments (Feio et al. 2023). Bending the curve of freshwater biodiversity loss has emerged as a global imperative, yet this objective remains highly challenging due to significant gaps in our understanding of biodiversity dynamics (Dudgeon and Strayer 2025). Current knowledge of biodiversity is heavily biased toward charismatic organisms, most of which are vertebrates, leaving substantial gaps in understanding of invertebrate diversity, despite their dominance in global animal populations and their critical roles underpinning ecological processes (Duffus et al. 2023).

The family Chironomidae (non‐biting midges) is often the dominant component of benthic macroinvertebrate assemblages in aquatic systems and plays a pivotal role in supporting ecosystem functioning (Berg and Hellenthal 1992). To date, over 7000 chironomid species have been documented worldwide, with estimates suggesting thousands more remain undiscovered (Kirk‐Spriggs and Sinclair 2017). As one of the most ubiquitous insect groups, Chironomidae inhabit nearly all aquatic habitats across every continent, including Antarctica (Rico and Quesada 2013). Chironomidae have long been recognized as potential bioindicators of freshwater ecosystem health, owing to their high diversity and wide range of ecological tolerances (Nicacio and Juen 2015; Leszczyńska et al. 2019). Empirical data indicate that Chironomidae assemblages respond sensitively to both local and regional environmental gradients, including various aspects of water quality (e.g., nutrients, dissolved oxygen, and salinity), habitat substrate, catchment land use, and climatic conditions (Nicacio and Juen 2015; Kranzfelder et al. 2015; Matthews‐Bird et al. 2016). However, Chironomidae are often excluded from biodiversity monitoring and ecological assessments due to difficulties in morphological identification (Chimeno et al. 2023), despite exhibiting substantially greater species richness compared to other bioindicator groups such as Ephemeroptera, Trichoptera, and Plecoptera (Seymour et al. 2021).

DNA barcoding has helped alleviate taxonomic identification challenges by linking morphological identifications with standardized DNA sequences (Hebert et al. 2003), typically a ~658‐bp fragment of the Cytochrome c Oxidase Subunit I (COI) gene used for animal taxa. In Chironomidae, DNA barcoding has been widely incorporated into integrative taxonomic frameworks to uncover cryptic species (Han et al. 2021; Lin et al. 2018; Makarchenko et al. 2023) and to associate different life stages (Stur and Ekrem 2011; Krosch and Cranston 2012). DNA barcodes generated through taxonomic research serve as foundational data for comparable ecological assessments (Blattner et al. 2025), biogeographic studies (Seymour et al. 2024), and metabarcoding‐based biodiversity surveys (Seymour et al. 2021; Taberlet et al. 2012). With over 1.7 million publicly accessible barcode records, Chironomidae are among the most extensively sequenced insect families in the Barcode of Life Data System (BOLD; accessed August 2025). However, a recent evaluation of the global Chironomidae barcoding library revealed significant biases in geographic and taxonomic representation, as well as notable deficiencies in the accuracy and resolution of associated taxonomic annotations (Han et al. 2023). Assignment of Linnaean species names to DNA barcodes requires long‐term morphological scrutiny, which is complicated by the high prevalence of cryptic diversity and synonymy in Chironomidae (Šamulková et al. 2025). Consequently, molecular operational taxonomic units (MOTUs) derived from DNA barcode clustering have been proposed as proxies for species in biodiversity assessment, as well as in ecological and biogeographic studies (Seymour et al. 2024; Pentinsaari et al. 2020).

The distribution of Chironomidae species warrants further investigation within the metacommunity framework, as biological communities are increasingly recognized as interconnected components of a regional species pool rather than isolated entities (Chase et al. 2020). Metacommunity theory hypothesizes that biological communities are structured by fundamental ecological processes including environmental filtering, biotic interaction, dispersal, and ecological drift (Thompson et al. 2020; Chase et al. 2020). Chironomid assemblages are often linked to environmental drivers in ecological research, but dispersal‐related spatial factors often are understudied despite documented evidence of downstream drift in larval and pupal stages and overland dispersal as winged adults (Armitage et al. 1995; Krosch et al. 2011a). The dual dispersal pathways of Chironomidae indicate that their distribution is likely influenced by the interplay of terrestrial landscape characteristics and hydrological dynamics (Delettre and Morvan 2000; Milošević et al. 2022). Elucidating the relative contributions of ecological processes in shaping metacommunity dynamics is essential for effective bioassessment, as dispersal processes may modulate the response pattern of indicators to environmental changes (Leibold et al. 2004; Milošević et al. 2022).

Hong Kong, a densely populated megacity in southern coastal China, features rugged topography and high precipitation, fostering dense lotic systems. Recent assessments reveal that Hong Kong's biodiversity is under increasing anthropogenic pressure, with 46% of freshwater fish and 14% of dragonfly species at risk of local extinction (Or and Chan 2025). While high insect diversity has been well documented for groups such as butterflies and dragonflies (Dudgeon and Corlett 2011), research on Chironomidae remains limited in Hong Kong, with only 17 recorded species to date (Lau 2025). To address this knowledge gap, this study presents the first systematic survey of Chironomidae diversity in Hong Kong. Our specific objectives are: (1) to update the regional species inventory and establish a well‐curated DNA barcode reference library for Hong Kong Chironomidae; (2) to elucidate the mechanisms underlying metacommunity assembly in Hong Kong Chironomidae, explicitly considering dispersal processes; and (3) to advance understanding of the global distribution patterns and biogeography of Chironomidae through cross‐database matching between our local reference library and public barcode repository.

## 2 Materials and Methods

### 2.1 Study Area and Chironomidae Sampling

**FIGURE 1:**
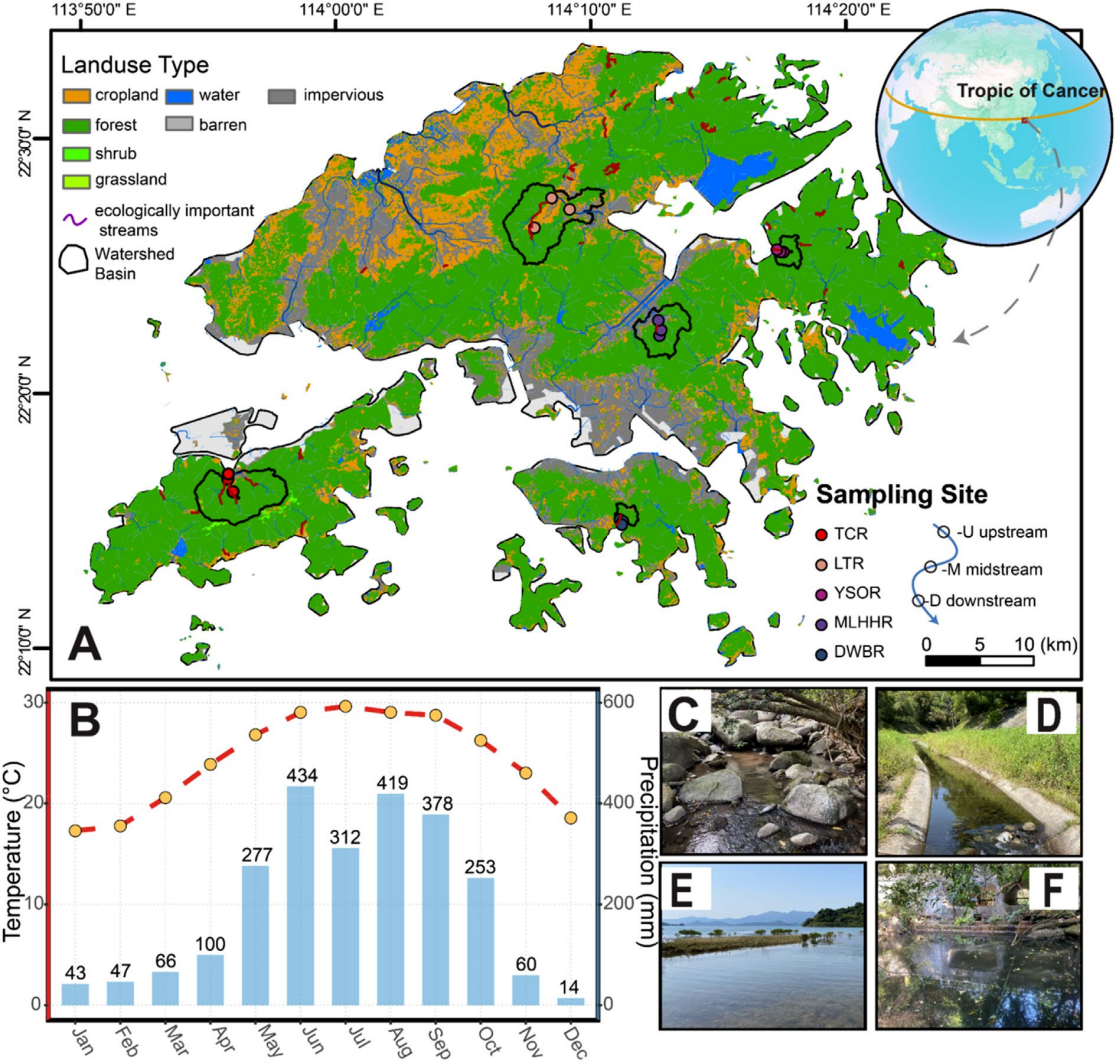
Geographic and climatic overview of the study area. (A) Geographic location (red points) and land use composition of Hong Kong, and the spatial distribution of sampling sites (black outlines depicting the drainage area of each study site). (B) Long‐term monthly mean air temperature and total precipitation (2015–2024). (C–F) Representative habitat types at the sampling sites: (C) headwater stream, (D) urbanized channel, (E) estuarine zone, (F) small inland pool. Climatic data were retrieved from the Hong Kong Observatory (https://www.hko.gov.hk/sc/index.html).

Hong Kong lies on the southern coast of China (22°09′ N–22°37′ N) and covers a land area of ~1110 km^2^ (Dudgeon and Corlett 2011). The territory encompasses mainland areas and over 200 outlying islands (Figure 1A). Abundant precipitation and rugged terrain have shaped dense riverine networks. Hong Kong experiences a subtropical monsoon climate, with 75.7% of annual precipitation concentrated between May and September (Figure 1B). Unlike most comparable latitudes, Hong Kong exhibits greater seasonal temperature variation, ranging from 17.3°C in January to 29.6°C in July.

To characterize chironomid fauna in Hong Kong, we selected five stream sections with broad geographic coverage as sampling sites (Figure 1A): Tung Chung River (TCR), Lam Tsuen River (LTR), Yung Shue O River (YSOR), Mai Lai Hau Hang River (MLHHR), and Deep Water Bay River (DWBR). The five streams overlap with ecologically important streams identified by the Agriculture, Fisheries and Conservation Department (AFCD) of Hong Kong for their high conservation value. Within each stream, three longitudinal sampling sites were established to capture habitat heterogeneity, including headwaters, urban channels, estuaries, and ponds (Figure 1C–F). The exact locations, including site codes and geographic coordinates, are provided in Table S1.

Adult chironomids were collected biweekly using Malaise traps placed along the banks of each stream (*n* = 15 sites), with traps operating continuously over 12 months. Each trap was equipped with a 500 mL collection bottle containing 80% ethanol as a preservative. Pupae (i.e., pharate adults) and larvae were collected quarterly from 200 m stream reaches at each site using a D‐net (250 μm mesh). All samples were manually sorted under a dissecting microscope (Leica S9E, Germany), transferred to 2 mL microcentrifuge tubes filled with 80% ethanol, and stored at −20°C for morphological identification and molecular analysis.

### 2.2 Morphological Identification

For each sample, male adult and pupal specimens were first sorted into morphotypes under a stereomicroscope (Leica S9 E, Germany) based on distinct morphological features (e.g., body size, color pattern, wing setation, and genital morphology). We then selected 1–10 individuals (depending on counts) for slide mounting in Euparal (Carol Roth, Germany) under glass coverslips, following standard protocols (Sæther 1980). Morphological identifications were performed under a DM500 compound microscope (Leica, Germany) using reference taxonomic literature and taxonomic keys (Ekrem 2002; Han et al. 2020; Liu et al. 2022; Niitsuma 2013; Tang and Niitsuma 2017; Wang 1995; Wiederholm 1986; Zhang et al. 2016). Specimens that could not be identified to species were assigned provisional codes (e.g., *Polypedilum* sp. HK1) for subsequent analyses. After initial batches of slide‐mounted identifications, species with distinguishable features under the stereomicroscope were not repeatedly processed from other samples within the same site to reduce workload. Morphotype sorting of larvae under a stereomicroscope is highly challenging; therefore, specimens were initially assigned to subfamilies based on morphological features of eye‐spot and head capsule. Subsequently, 5–10 larval specimens from each subfamily were randomly selected for slide mounting and identified to genus level using a compound microscope and taxonomic identification keys (e.g., Andersen et al. 2013; Cranston and Tang 2024). This workflow was applied independently for each site to ensure accurate estimation of species richness. Morphological identification was primarily carried out by the first author, with verification and cross‐checking performed by co‐authors and external taxonomic experts specializing in Chironomidae. Voucher slides are deposited in the Environmental DNA and Environmental Ecology Laboratory, School of Biological Sciences, The University of Hong Kong.

### 2.3 DNA Barcoding

When available, at least three identified specimens from each morphological species were selected for DNA barcoding to capture intraspecific genetic variation (Fontes et al. 2021). The thorax of individual specimens was excised for DNA extraction. Genomic DNA was extracted using the MAGEN Tissue DNA Kit (Beijing Science and Beyond Technologies, China), following the manufacturer's instructions. The standard Cytochrome c Oxidase Subunit I (COI) barcode fragment was amplified using universal primers (LCO1490/HCO2198; Folmer et al. 1994). Specimens that failed initial amplification were re‐amplified using a custom reverse primer (440R: 5′‐TGACCAAAAAATCAAAATAA‐3′) in combination with Folmer's forward primer, yielding a shorter 630‐bp amplicon. DNA amplification was performed in 25 μL reactions containing 12.5 μL of 2× Hieff PCR Master Mix (Yeasen, China), 0.5 μL of each 10 μM primer, 3 μL template DNA, and 9.5 μL ddH_2_O. Amplification was carried out on a PTC Tempo Thermal Cycler (USA) using the following program: initial denaturation at 95°C for 5 min; 35 cycles of denaturation at 94°C for 30 s, annealing at 49°C for 30 s, and extension at 72°C for 1 min; followed by a final extension at 72°C for 10 min. PCR products were verified by 1% agarose gel electrophoresis and then sent to Genewiz Biotechnology (Suzhou, China) for purification and bidirectional Sanger sequencing.

Raw sequences were processed using Geneious Prime (version 2024.0.5). Forward and reverse reads were assembled using the De Novo Assemble tool, with PCR primers and low‐quality regions (error probability > 0.05) trimmed. Multiple sequence alignment was performed using the MUSCLE algorithm (Edgar 2004), and indels were manually removed. All sequences were then translated into amino acids to confirm the absence of premature stop codons, ensuring they represented functional protein‐coding regions. Subsequently, each barcode was queried against the NCBI nucleotide (*nt*) database using BLAST (https://blast.ncbi.nlm.nih.gov/Blast.cgi) to screen for potential contamination. The final DNA barcode sequences, along with their corresponding trace files and associated metadata, have been deposited in the BOLD system under dataset DS‐CHIOHK (DOI: https://doi.org/10.5883/DS‐CHIOHK) and in GenBank under accession numbers PX554527–PX555354.

### 2.4 Identification Validation and Barcode Analysis

Morphological identifications were confirmed as valid, based on DNA barcode analysis. A neighbor‐joining phylogenetic tree was constructed in BOLD (Taxon ID Tree) using the Kimura 2‐parameter (K2P) model. Morphospecies showing high intraspecific genetic distances or unexpected phylogenetic placements (e.g., paraphyly, polyphyly, or unusually long branches) were re‐examined morphologically for potential contamination, mislabeling, or identification error. This integrative approach ensured strong concordance between morphological and genetic evidence for all delimited species, although some identification errors do remain possible (Han et al. 2023). The optimal threshold (OT) and barcoding efficiency (BE) were determined in the R package spider (Brown et al. 2012). The OT is defined as the genetic distance threshold range that minimizes identification errors, while BE represents the proportion of accurate identifications achieved at the corresponding threshold (Gadawski et al. 2022).

Two species delimitation methods based on genetic distance were also conducted to compare with the morphological result. Barcode Index Numbers (BIN) were generated on BOLD using the Refined Single Linkage (RESL) algorithm (Ratnasingham and Hebert 2013). Assemble Species by Automatic Partitioning (ASAP) is an algorithm that performs hierarchical clustering based on pairwise genetic distances and generates ranked partition schemes according to composite scores (Puillandre et al. 2021). These scores integrate probabilities of groups representing panmictic species with barcode gap width metrics. ASAP analysis was conducted on the online platform using default parameters, except for the selection of K2P genetic distance (https://spartexplorer.mnhn.fr/delimitation).

### 2.5 Cross‐Database Barcode Matching Analysis

After removing duplicate sequences, the newly generated barcode dataset was queried against the public animal barcode library in the BOLD database using BOLDigger software (Buchner and Leese 2020), with the “Genus and Species Search” mode enabled. Given that intraspecific COI barcode divergence in Chironomidae typically ranges from 0.04 to 0.09 (Gadawski et al. 2022; Han et al. 2021; Song et al. 2018), we adopted a more conservative approach by retaining only matches with a similarity threshold > 0.97 to ensure high‐confidence species‐level identifications. To evaluate the robustness of the result, parallel analyses were performed using more stringent thresholds ranging from 0.98 to 1.00. Metadata for matched sequences were retrieved from BOLD (accessed August 2025). For records lacking precise geographic coordinates, approximate latitude and longitude were estimated from textual locality descriptions using Google Maps.

### 2.6 Abiotic Variable Collection

We measured six water‐quality parameters monthly using a multiparameter probe (Horiba U‐52, Japan), including water temperature (°C), dissolved oxygen (mg/L), pH, conductivity (mS/m), turbidity (NTU), and salinity (ppt). Surface flow velocity was measured monthly with a water flow meter (HYDRO‐BIOS, Germany). At each sampling site, substrate composition was visually assessed and categorized into five types: silt, sand, gravel, cobble, and boulder. Watershed areas were calculated from digital elevation models using ArcGIS v10.1 (Environmental Systems Research Institute Inc.). Land use composition within each watershed was summarized as the percentage cover of land cover classes, including cropland, forest, shrubland, grassland, water, barren land, and impervious surfaces, based on a published 10 m resolution land cover dataset (Gong et al. 2019). Furthermore, we calculated the Human Activity Intensity of Land Surface (HAILS) as a proxy for anthropogenic pressure by converting land use classes into construction‐land equivalents using class‐specific coefficients (Xu et al. 2016).

Given that Chironomidae disperse both as aquatic immature stages within stream networks and as flying adults over terrestrial landscapes, we modeled spatial structure using two complementary eigenfunction approaches that reflect these distinct dispersal mechanisms. Distance‐based Moran's eigenvector maps (dbMEM) analysis was implemented to capture symmetric spatial autocorrelation (Legendre and Gauthier 2014), and asymmetric eigenvector maps (AEM) to represent directional processes along the river network (Blanchet et al. 2008a). dbMEM constructs a spatial weighting matrix from the sampling configuration and extracts a set of orthogonal spatial eigenvectors ordered from broad to fine spatial scales, thereby describing multi‐scale, direction‐agnostic spatial structure (Legendre and Gauthier 2014). In contrast, AEM derives eigenvectors from a directed connectivity matrix and thus encodes explicit directionality in connectivity (Blanchet et al. 2008a).

Spatial predictors were generated using *dbMEM* and *AEM* functions in the R package “adespatial” (Dray et al. 2025). Only spatial eigenvectors associated with positive eigenvalues were retained to focus on positive spatial autocorrelation and interpretable spatial structures. The spatial eigenvectors that were strongly correlated with any environmental variables (|Pearson *r*| > 0.7) were removed to isolate spatial effects from environmental gradients. The resulting dbMEM and AEM vectors were then used as spatial predictors in subsequent variance partitioning analyses.

Ecological tolerances of matched species to climatic conditions in the cross‐database barcode matching analysis were assessed using extracted mean annual precipitation and mean annual minimum monthly temperature for each matched sample site using ArcGIS v10.1 (Environmental Systems Research Institute Inc.), based on georeferenced metadata from the BOLD database. Climatic data were obtained from WorldClim at a 30 m spatial resolution for the period 2010–2019 (Fick and Hijmans 2017) and averaged over the 10‐year period.

### 2.7 Statistical Analysis

#### 2.7.1 Diversity and Community Assembly Drivers of Hong Kong Chironomidae

All data analyses and visualizations were conducted in R v4.5.1. (R Core Team 2025). Species count data were converted to presence–absence format, and environmental variables were standardized using the *decostand* function in the R package “vegan” (Oksanen et al. 2022). Diversity was assessed at three hierarchical levels: alpha diversity (species richness per sampling site), beta diversity (community dissimilarity among sites), and gamma diversity (cumulative species richness within a single stream or across all sites). Beta diversity was calculated as Sørensen dissimilarity and partitioned into turnover and nestedness using the *beta.pair* function in the R package “betapart” (Baselga and Orme 2012). Partial Mantel tests were performed to examine relationships between diversity index (alpha and beta diversity) and local environmental factors in the R package “vegan” (Oksanen et al. 2022), with geographic distance matrices included as a conditioning variable to account for spatial autocorrelation. Changes in beta diversity and its components along spatial and environmental gradients (i.e., Euclidean distance) were assessed with linear regression models.

Principal coordinates analysis (PCoA) was performed on a Jaccard dissimilarity matrix to visualize among‐site differences in community composition using the R package “ape” (Paradis and Schliep 2019). Differences in community composition among streams were tested using analysis of similarities (ANOSIM) with 999 permutations in the R package “vegan” (Oksanen et al. 2022). To model the response of community composition to environmental and spatial predictors (i.e., AEMs and MEMs), we conducted distance‐based redundancy analysis (dbRDA). Prior to analysis, environmental variables were screened for multicollinearity using variance inflation factors (VIF < 10). Forward selection was implemented using ordiR2step function in the “vegan” package, based on the double‐stopping criterion (the usual alpha significance level and the adjusted *R* ^2^ exceeding the global model), as described in Blanchet et al. (2008b). The independent effects of individual predictors were assessed via hierarchical partitioning with the “rdacca.hp” package (Lai et al. 2022).

#### 2.7.2 Large‐Scale Distribution Pattern Revealed by Cross‐Database Barcode Matching

To investigate the biogeographic affinities of Hong Kong, matched DNA barcode records were aggregated at both national and subnational (provincial or state) levels. Regional species richness was calculated based on taxonomic identifications from the local reference database. Geographic and climatic characteristics associated with these matched barcodes were summarized at the species, genus, and subfamily levels to estimate the geographic and climatic extent occupied by Chironomidae taxa. Differences in precipitation and temperature distributions across Chironomidae subfamilies were evaluated using two‐sided Mann–Whitney *U* tests.

Each matched barcode was assigned to a climatic zone based on its geographic coordinates and to a biogeographic realm according to the classification scheme of Holt et al. (2013). To assess potential bias due to uneven barcode representation across regions, the relationship between public DNA barcode availability and regional species richness was visualized using LOESS regression, implemented via the *geom_smooth* function in the “ggplot2” package (Wickham 2010).

## 3 Result

### 3.1 Overview of Species Richness and DNA Barcode Library

**FIGURE 2:**
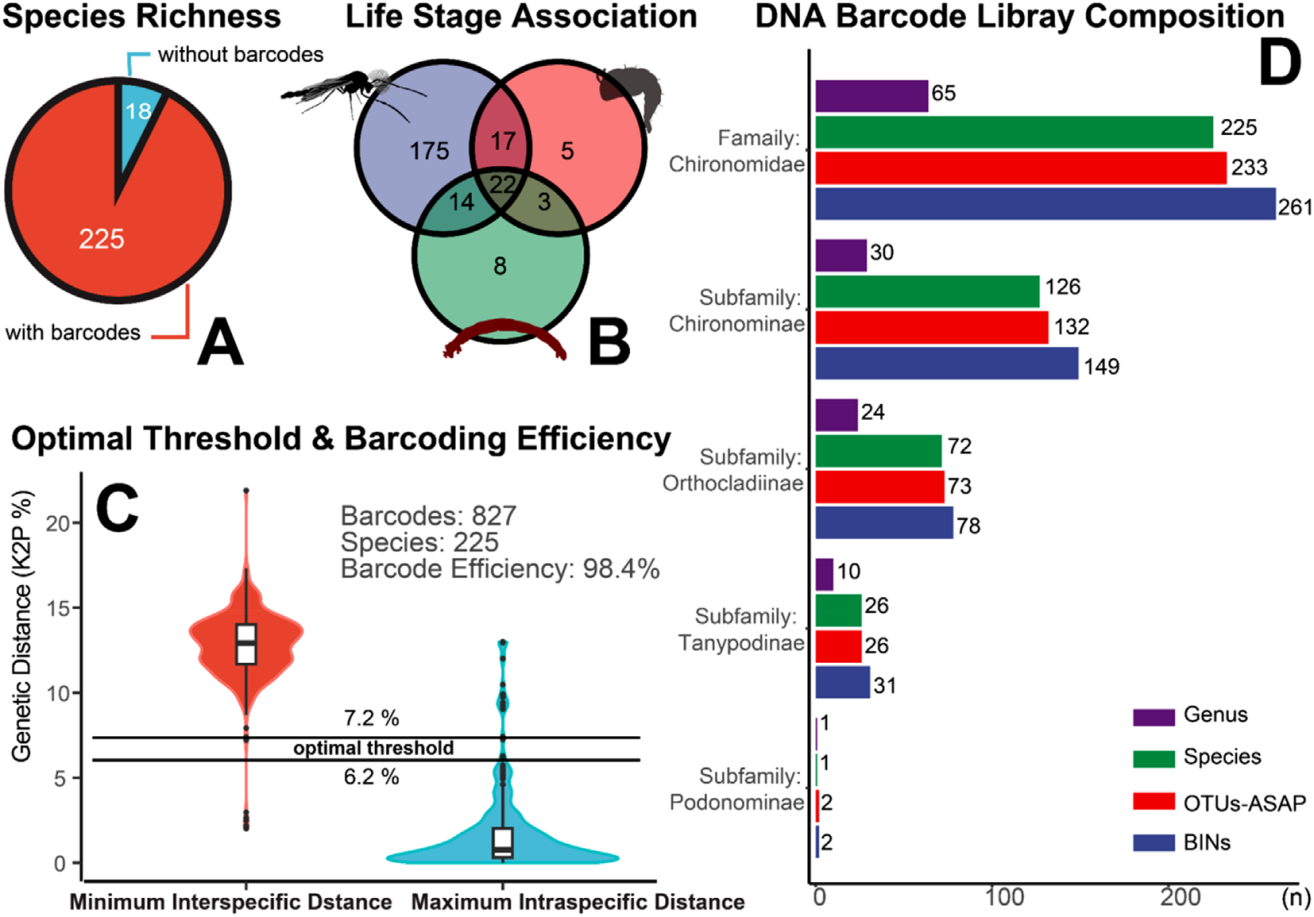
Summary of Chironomidae species richness and curated DNA barcode library. (A) Total species richness recorded and the number of species with DNA barcodes. (B) Life stage associations inferred through DNA barcoding or morphological examination on pupae (pharate adults). (C) Distribution of minimum interspecific and maximum intraspecific genetic distances (K2P distance) in the curated DNA barcode library. The dark horizontal line indicates the optimal threshold interval for species delimitation. (D) Taxonomic composition of DNA barcode library at subfamily level. “Species” refers to taxa identified using combined morphological and phylogenetic analyses (Neighbor‐Joining tree); “OTUs‐ASAP” denotes molecular operational taxonomic units proposed by the ASAP software; “BINs” refers to Barcode Index Numbers generated in the BOLD Systems database.

Integrative taxonomic analysis of 1725 mounted specimens (1119 male adults, 117 pupae, and 489 larvae) revealed 243 species across 65 genera and four subfamilies (Figure 2A). Of these, 827 specimens yielded high‐quality DNA barcodes, representing 225 species (Figure 2A). Notably, 240 species, 56 genera, and one subfamily (Podonominae) were newly recorded in Hong Kong (Table S2). DNA barcoding combined with morphological examination enabled the association of 39 pupal and 36 larval morphospecies with male adults. No barcoding gap was observed between minimum interspecific (12.8% ± 2.1%) and maximum intraspecific genetic distances (1.7% ± 2.5%). The highest intraspecific distance was observed in *Polypedilum cultellatum* , with a maximum of 6.09% between two clusters that have subtle morphological differences in the superior volsella of the hypopygium. *Cricotopus* sp. “8CSG” and *C. bimaculatus* displayed minimal interspecific divergence (2.35%), but they exhibit consistent morphological distinctions in abdominal color patterns. Overall, barcoding efficiency remained high (98.4%) at the optimal threshold (Figure 2C). In terms of species richness, Chironominae and Orthocladiinae were dominant, whereas Podonominae was represented solely by *Paraboreochlus okinawanus* (Figure 2D). Species delimitation based on genetic divergence generated 261 BINs in the BOLD system and 233 OTUs in the ASAP software, slightly exceeding the 225 species identified through an integrative approach combining morphology and phylogeny.

### 3.2 Alpha and Gamma Diversity

**FIGURE 3:**
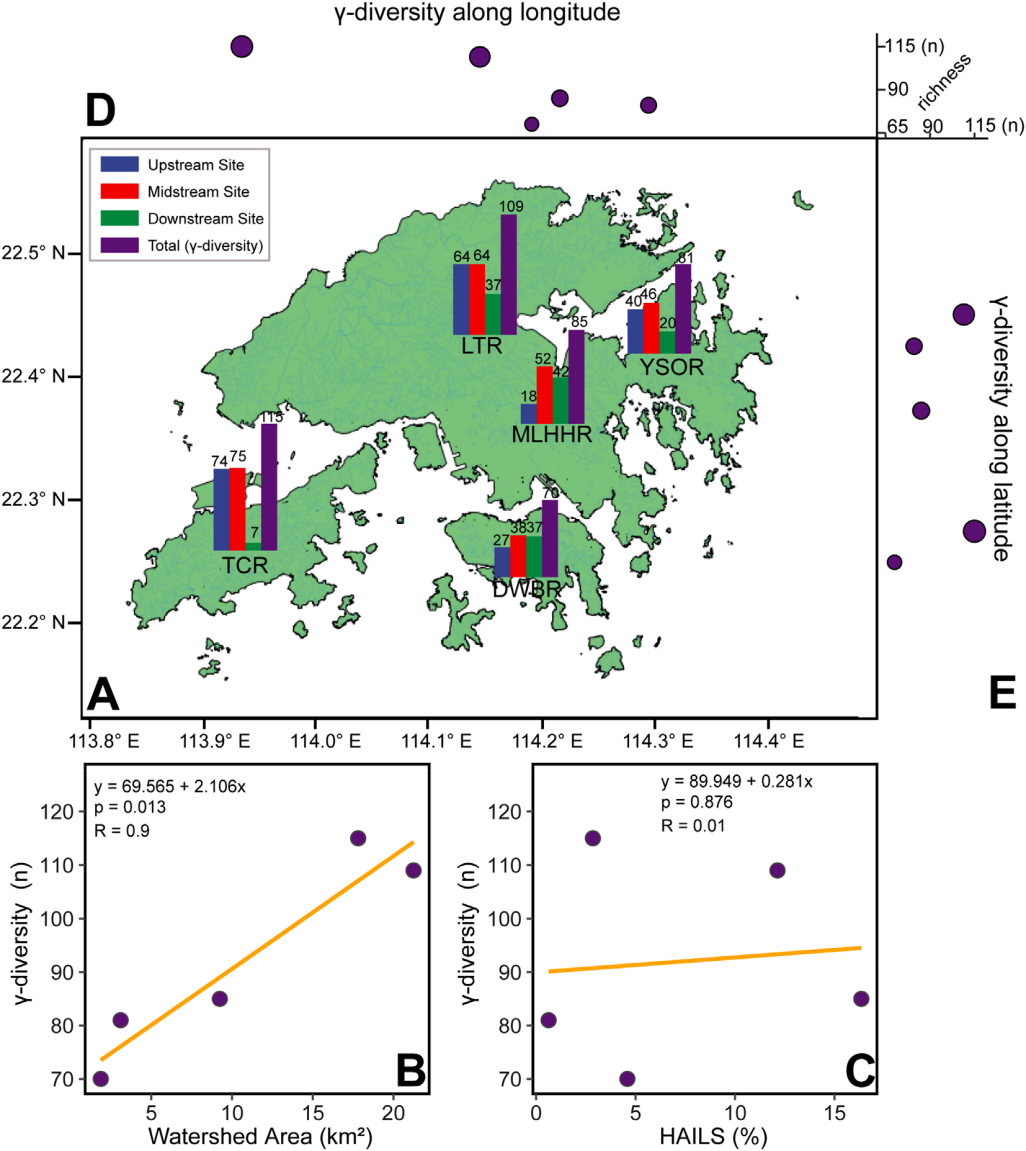
Spatial pattern of alpha‐ and gamma‐diversity. (A) Bar plots indicate local species richness (alpha‐diversity) at individual sampling sites and cumulative richness (gamma‐diversity) per stream. (B) Relationship between gamma‐ diversity and watershed area. (C) Relationship between gamma‐ diversity and Human Activity Intensity of Land Surface. Variation in gamma‐diversity across latitude (D) and longitude (E).

At the watershed scale, cumulative species richness (gamma‐diversity) displayed no latitudinal pattern but a subtle east–west gradient (Figure 3D,E), with three eastern streams (DWBR, MLHHR, YSOR) exhibiting lower richness than western streams (TCR, LTR). Gamma‐diversity exhibited a strong linear relationship with watershed area (*R* = 0.9, *p* = 0.013, Figure 3B) but showed no association with the human activity intensity index calculated based on land use composition (Figure 3C). Specifically, TCR had the highest richness (*n* = 115), while another island stream in Hong Kong Island, DWBR, exhibited the lowest (*n* = 70) richness. Species richness was particularly low at two estuarine sites, with only 7 and 20 species recorded at TCR‐D and YSOR‐D, respectively. Despite the brackish conditions, some adults of typically freshwater species were occasionally detected at these two sites, such as *Ablabesmyia daiensis*, *Kiefferulus tainanus*, and *Microtendipes umbrosus*. The urban channel site (LTR‐D), characterized by a cement substrate and high‐velocity water flow, harbored 37 species, approximately half the species diversity found in its more natural upper reaches. After controlling for geographic distance, the partial Mantel test suggested that species richness significantly decreased with salinity (Mantel *r* = −0.31, *p* = 0.05) yet had no strong relationship with other local environmental variables (Figure S1A).

### 3.3 Beta Diversity, db‐RDA Model and Variation Partitioning

**FIGURE 4:**
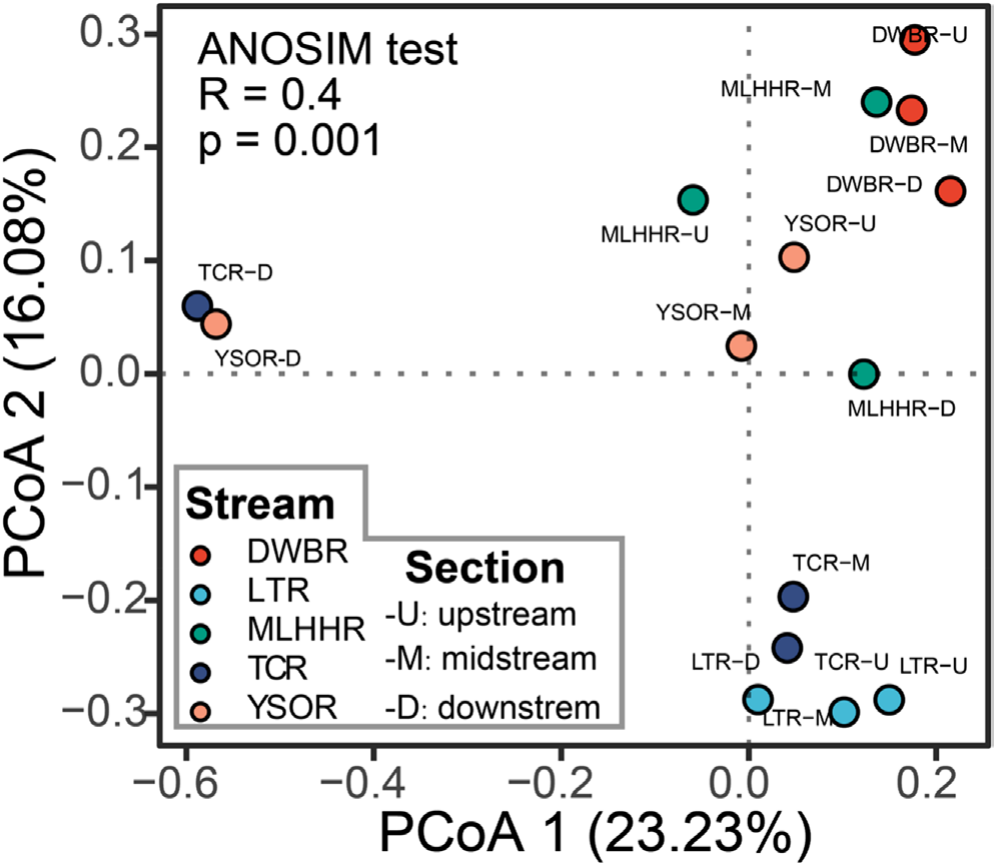
Principal coordinates analysis (PCoA) of Chironomidae community composition across 15 sampling sites based on the Jaccard dissimilarity matrix. Points are colored by stream identity. ANOSIM test shows significant differences in species composition among streams with 999 permutations.

The ANOSIM test revealed that Chironomidae assemblages within the same stream were more similar to each other than those across different streams (*R* = 0.4, *p* = 0.01; Figure 4). The two estuarine sites (TCR‐D and YSOR‐D) arranged at the negative end of Axis 1 (23.23% variance) of PCoA with great separation from other freshwater sites (Figure 4; upper left quadrant). Partial mantel tests showed strong associations between community dissimilarity and five local environmental variables (Figure S1B; Table S3): water temperature, salinity, flow velocity, sand content, and boulder cover.

**FIGURE 5:**
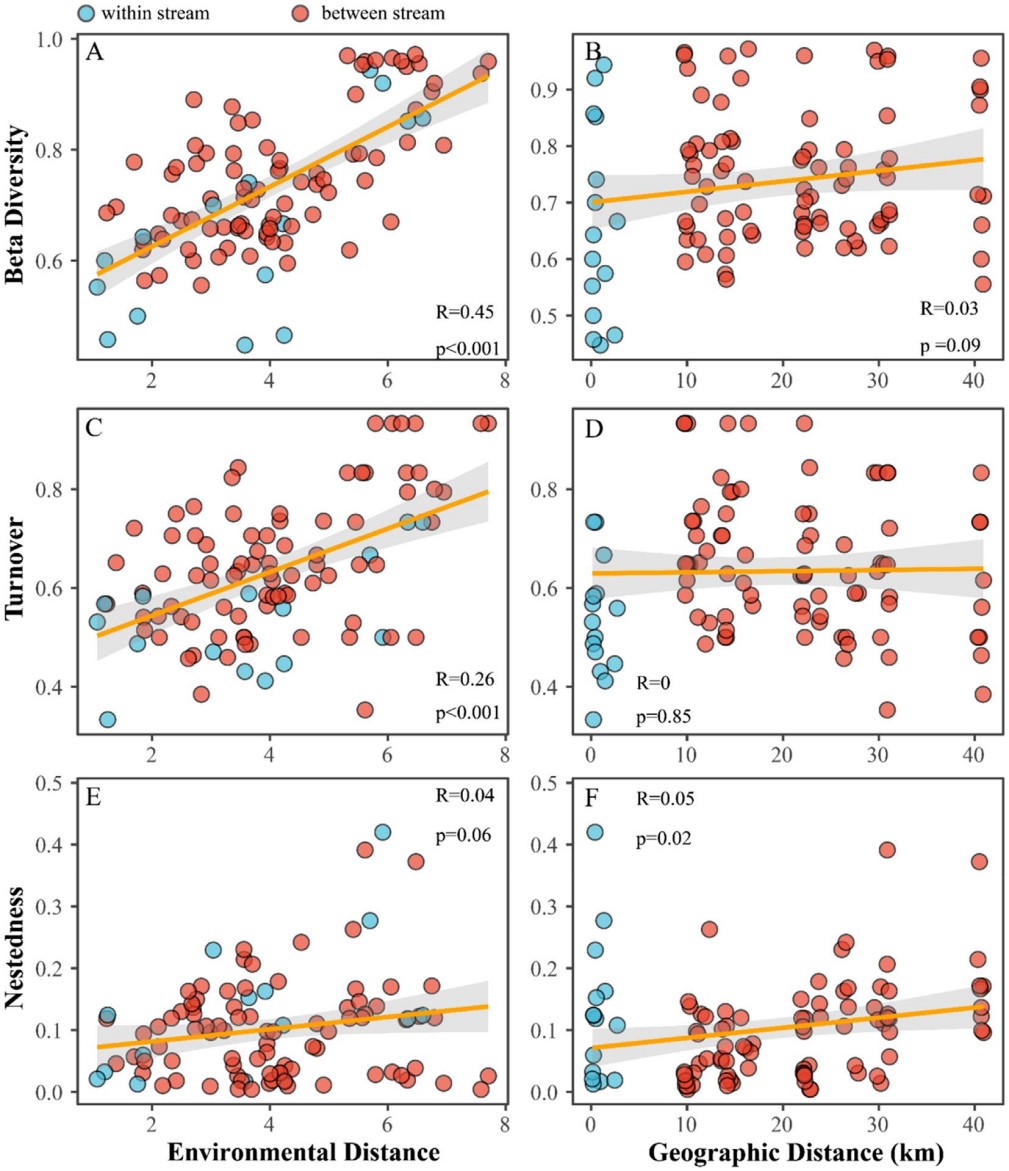
Beta diversity, its partitioned components, and their variation with environmental or geographic distance. (A, C, E) Relationships between beta diversity and environmental distance. (B, D, F) Relationships between beta diversity and geographic distance.

**FIGURE 6:**
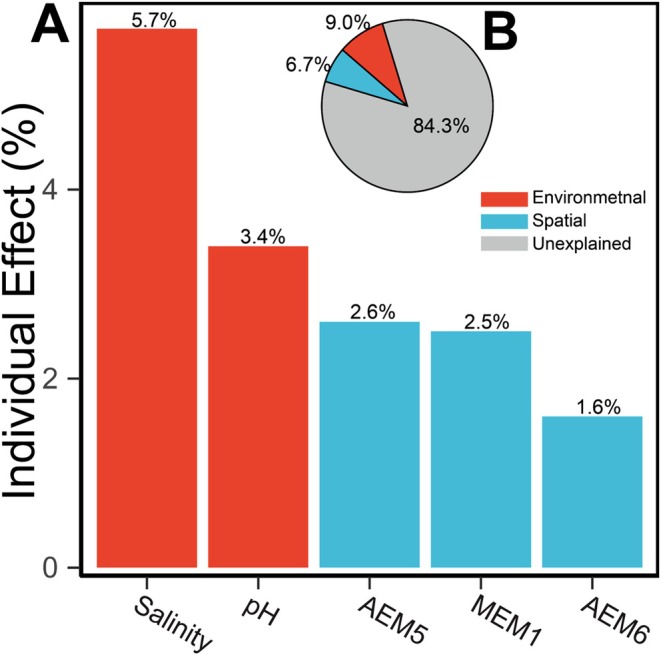
Hierarchical partitioning (HP) analysis of the contributions of key explanatory variables selected by forward selection from separate environmental and spatial datasets. (A) Independent effects of each variable. (B) Total explanatory contribution of each variable category.

Community dissimilarity was strongly associated with local environmental conditions but not with geographic distance (Figure 5A,B). Specifically, beta diversity increased significantly with environmental distance, driven predominantly by species turnover rather than nestedness (Table S4). Turnover itself was positively correlated with environmental gradients but unrelated to spatial distance, whereas nestedness showed only weak associations with both (Figure 5C–F). Separate forward selection identified salinity and pH as key environmental predictors (adjusted *R* ^2^ = 12.5%; Figure S2A) and MEM1, AEM5, and AEM6 as spatial predictors (adjusted *R* ^2^ = 10.7%; Figure S2B). In a joint db‐RDA model, these five variables together explained 15.7% of community variation (Figure S2C). Hierarchical partitioning attributed 9.0% of variation to environmental predictors, slightly exceeding spatial predictors (6.7%; Figure 6B). Salinity emerged as the single strongest predictor (5.7%), followed by pH and spatial eigenvectors (Figure 6A).

### 3.4 Fauna Comparison Between Local Fauna With Public Database

**FIGURE 7:**
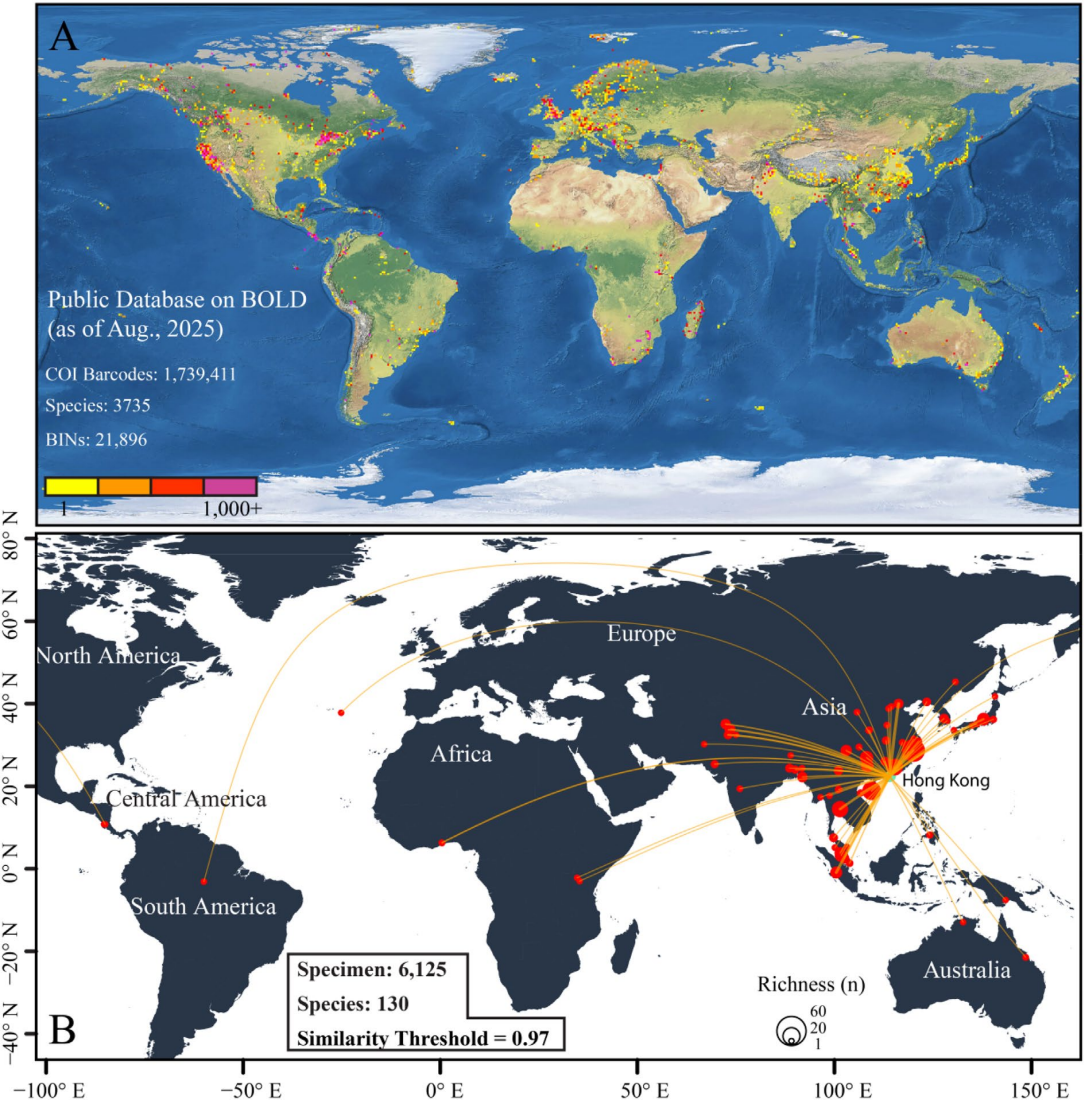
Cross‐database barcode matching analysis between local and global DNA barcode datasets. (A) Global contribution for DNA barcodes of Chironomidae in the public database of the BOLD system (as of August 2025). (B) Spatial distribution pattern of matched species based on DNA barcode similarity (97% similarity threshold). Matched occurrence records were aggregated at the provincial or state level and linked to Hong Kong with yellow lines. The size of red circles is proportional to the number of shared species.

**FIGURE 8:**
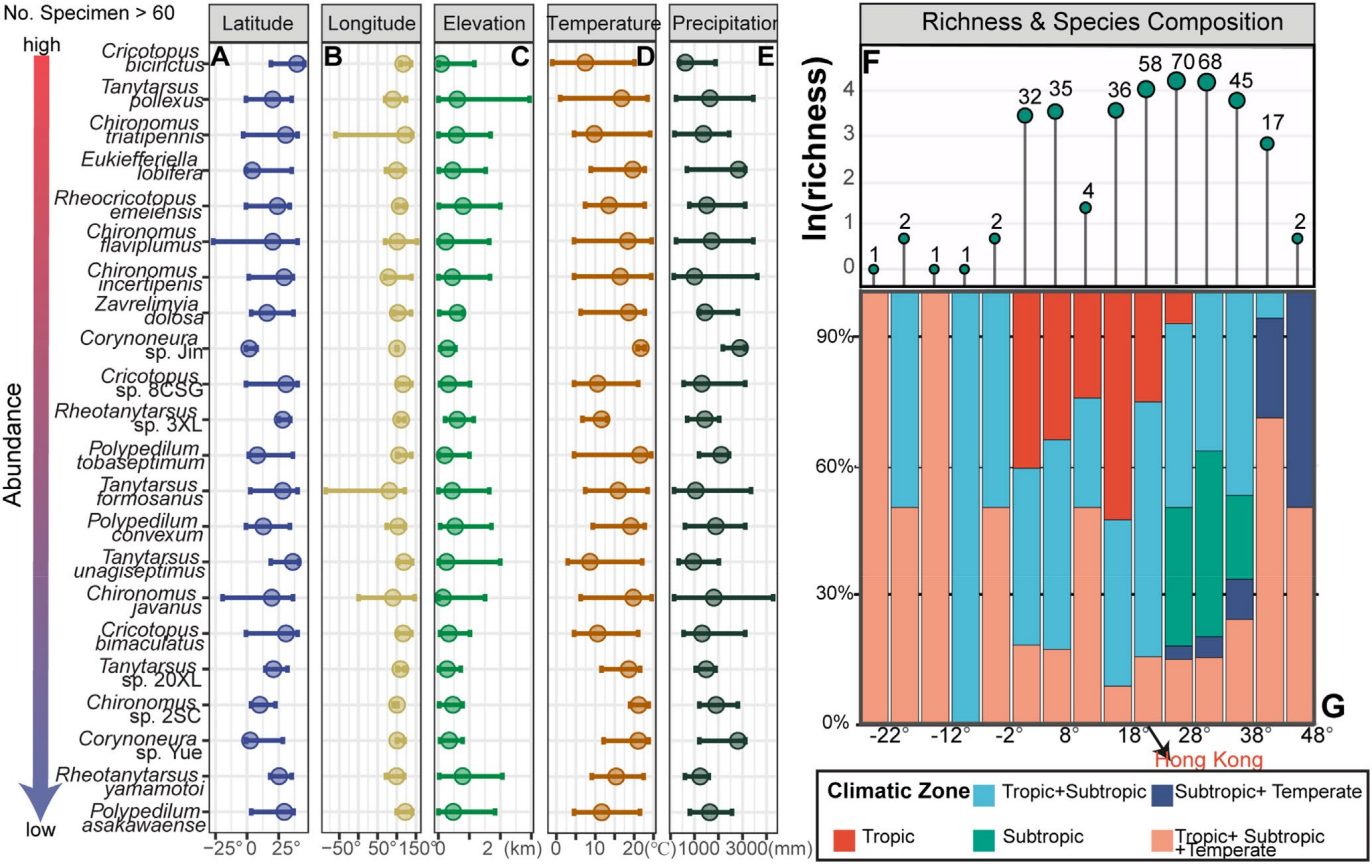
Geographic and climatic distribution ranges of chironomid species. Left panels: Distribution of common species (with > 60 specimens) across key geographic (A–C) and climatic variables (D, E). Right panels: Variation in species richness (F) and composition of climatic types (G) along the latitudinal gradient. Each species was assigned to a single climatic zone based on the geographic distribution of its specimens.

The Barcode of Life Data (BOLD) system hosts 1,739,411 Chironomidae DNA barcodes, encompassing 3735 species and 21,896 Barcode Index Numbers (BINs), with a notable distributional bias toward North America, Europe, and East and South Asia (Figure 7A). Of the 546 local Chironomidae barcodes, 358 (65.6%) matched public sequences in BOLD at ≥ 97% similarity, representing 130 species with georeferenced records. These matches spanned a broad geographic extent (27° S–45° N; 85° W–100° E) and were mainly concentrated in East and Southeast Asia (Figure 7B). Sporadic distributions were found in some distant regions, including Africa, Australasia, Europe, and Central and South America. Broad geographic distributions were even evident at the species level among the most commonly recorded Chironomidae species (*n* > 60), with mean latitudinal, longitudinal, and elevational ranges of 32.4° ± 13.7°, 57.2° ± 55.9°, and 409.3 ± 204.6 m, respectively (Figure 8A–C).

**FIGURE 9:**
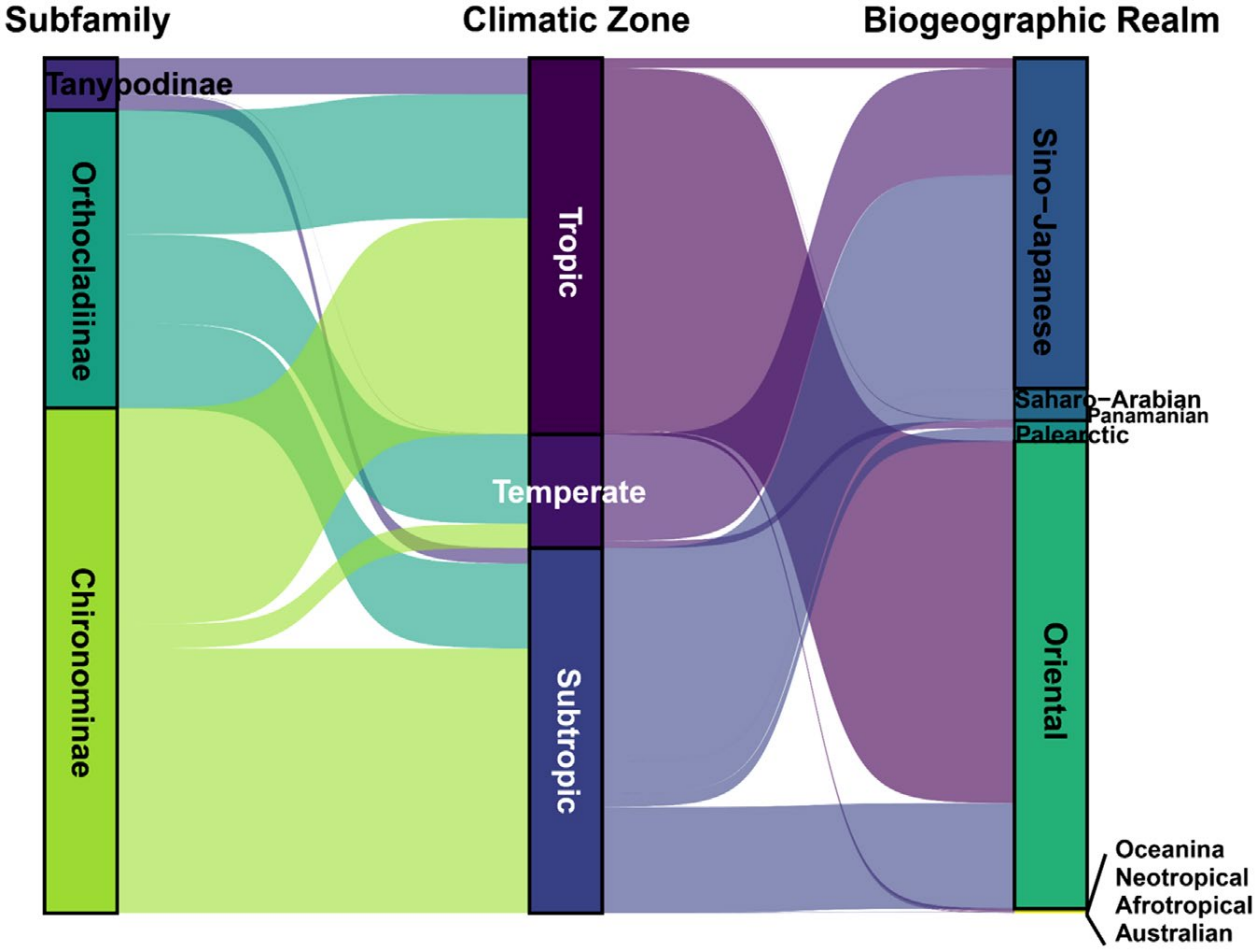
Sankey diagram illustrating the geographic distribution of matched DNA barcodes (0.97 similarity threshold). The left column displays specimen counts by subfamily, the middle by climatic zone, and the right by biogeographic realm (Holt et al. 2013). Flow widths are proportional to specimen numbers.

Species were assigned to climatic zones based on the geographic origins of matched specimens (Figure 8F,G). Over half (56.9%) were restricted to tropical (*n* = 37) or subtropical (*n* = 37) zones; the remainder showed broader distributions with 12 spanned tropical–temperate, four subtropical–temperate, and 40 tropical–subtropical zones. Most matched records originated from tropical (44.6%) and subtropical (42.6%) regions, with only 12.8% (835 records) from temperate areas (Table 1, Figure 9). Chironomidae genera exhibited broad climatic tolerances, especially those with higher species richness (Figure S3A–C). At the subfamily level, Chironominae and Tanypodinae showed more restricted distributions than Orthocladiinae, generally favoring warmer, drier habitats (Figure S3D). The matched species are predominantly found in regions with annual precipitation ranging from 1000 to 2000 mm and mean monthly minimum temperatures between 7°C and 24°C (Figure S4).

**TABLE 1:** Distribution of chironomid species and specimens across climatic zones and biogeographic realms.

	No. of species	No. of specimens
Climatic zone		
Tropical	89	2911
Subtropical	93	2775
Temperate	16	835
Biogeographic realm		
Oriental	90	3614
Sino‐Japanese	85	2454
Saharo‐Arabian	14	241
Palearctic	16	169
Australian	2	22
Afrotropical	1	7
Panamanian	1	5
Neotropical	1	5
Oceanian	1	4

*Note:* Species occur in multiple climatic and biogeographic zones and are counted separately for each zone. The updated zoogeographic framework proposed by Holt et al. (2013) was adopted.

Biogeographically, most records came from the Oriental (3614 records, 90 species) and Sino‐Japanese realms (2454 records, 85 species), with few from the Palearctic, Saharo‐Arabian, or other realms (Table 1, Figure 9). The Hong Kong chironomid assemblage was most similar to those in East and Southeast Asia (Figure S5). Raising the barcode similarity threshold to 100% retained this pattern but reduced matches to 1025 specimens (73 species), eliminating links to Australasia and the Americas (Figure S6). Match frequency did not correlate with regional barcode availability (Figure S7).

## 4 Discussion

Our study represents the first comprehensive assessment of Chironomidae biodiversity in Hong Kong, substantially increasing the known species richness from 17 to 243. The newly generated species list and DNA barcode library provide a foundation for continued taxonomic, ecological, and biogeographic research. Multivariate analyses revealed that environmental variables explained a greater share of community variation than spatial factors, with a substantial portion of the variation remaining unexplained. Cross‐database barcode matching analysis demonstrated that most species exhibit extensive geographic distributions and high climatic adaptability, alongside strong biogeographic affinities with East and Southeast Asia.

### 4.1 Chironomid Diversity: The Overlooked Majority

Despite their critical roles in freshwater ecosystems, the Chironomidae family is frequently excluded from routine biodiversity monitoring, resulting in significant knowledge gaps (Lencioni et al. 2018). The species richness of Chironomidae documented in this study (*n* = 243) across just five streams in Hong Kong is comparable to that of butterflies (*n* = 272) and approximately 1.8 times that of dragonflies, despite both groups having been surveyed extensively over multiple decades (Or and Chan 2025). This contrast highlights the importance of focusing on overlooked, non‐charismatic species that form the foundation of biodiversity and ecosystem functioning. As Chironomidae have strong potential as sentinels of ecosystem health, increased research to resolve their biodiversity dynamics would substantially advance both taxonomic knowledge and broader conservation efforts (Lencioni et al. 2018; Rossaro et al. 2022). As such, our species‐level DNA barcode library helps fill critical gaps in taxonomic coverage and geographic representation in public reference databases, thereby enhancing the reliability and ecological inference of eDNA‐based assessments of freshwater ecosystems in Hong Kong and surrounding regions.

The documentation of 37 species at the concrete‐lined channel site is noteworthy, as such habitats are generally expected to harbor low aquatic biodiversity dominated by pollution‐tolerant species (Gomes and Wai 2020). The unexpectedly high species richness observed here may, in part, reflect recent ecological restoration efforts undertaken by local authorities. Hong Kong encompasses approximately 200 watercourses, most of which were converted into nullahs or channelized between the 1970s and 2000s to mitigate flooding, though at great detrimental cost to biodiversity (Duan et al. 2022). In recent decades, however, great efforts have been made to restore biodiversity and ecological function of selected channel segments. For example, the reintroduction of natural substrates such as cobbles and boulders has created available microhabitats, with the intent to facilitate the colonization of aquatic macroinvertebrates (Vermonden et al. 2009). Conservation and recovery of aquatic invertebrates in these habitats may also benefit higher trophic levels (Eisenhauer and Hines 2021), as inferred from our frequent observations of fish and waterfowl during field sampling. However, full ecological surveys are needed to generate the baseline data required for adequate assessments across Hong Kong, which are currently severely lacking.

### 4.2 Assembly Mechanisms Underlying Spatial Patterns of Biodiversity

Our study showed that the distribution of Chironomidae species is associated with both environmental and spatial factors, aligning with common observations in riverine macroinvertebrate communities (Astorga et al. 2011; Heino and Korsu 2008; Milošević et al. 2022; Serra et al. 2017; Seymour et al. 2016). Despite the commonly reported influence of human activities on biodiversity, our study observed no notable impact, likely due to most sampling sites being situated along streams classified as ecologically significant by local authorities, which experience relatively low levels of human disturbance. Previous studies also suggested that weak anthropogenic signals can be obscured by natural environmental and spatial gradients (Heino et al. 2007; Ligeiro et al. 2013). Likewise, the weak correlations between alpha‐diversity and environmental factors, aside from salinity, may reflect the relatively limited range of environmental gradients in our study. Nonetheless, significant species turnover along environmental gradients was observed (Figure 5C), highlighting the considerable value of Chironomidae as sensitive bioindicators for subtropical streams (Rossaro et al. 2022; Serra et al. 2017).

Hierarchical partitioning analysis suggests that environmental variables predominate over spatial predictors in structuring chironomid metacommunities in Hong Kong. Salinity and pH were the two strongest drivers, whose impact on species composition of lotic Chironomidae communities have been reported in previous studies (Orendt 1999; Pinder 1986; Zinchenko et al. 2019). Notably, several environmental variables were strongly collinear, so the individual effects of salinity and pH may also capture variation linked to correlated factors (Dormann et al. 2013), including water temperature, flow velocity, dissolved oxygen, and substrate. Local environmental variation may reflect larger‐scale gradients (e.g., tidal dynamics and anthropogenic land‐use), whose complex interactions require validation through expanded spatial sampling and higher‐resolution data.

Dispersal limitation was minimal and likely masked by more dominant environmental filtering, as indicated by the weak distance‐decay pattern observed (Figure 5B; Brown and Swan 2010). This pattern aligns with observations in stream networks across small to intermediate spatial scales, where actively dispersing flighted taxa more readily track environmental variability (Grönroos et al. 2013; He et al. 2020; Heino and Mykrä 2008; My Heino et al. 2015; Tonkin et al. 2018). Here, mass effects may play a more important role than dispersal limitation for community assembly at small spatial scales (Heino et al. 2015). High‐rate dispersal events can lead to the occurrence of species in unsuitable environmental conditions and homogenize communities at neighboring sites (Leibold et al. 2004.), which is consistent with our observation of higher community similarity within streams than among streams. Mass effects have been documented for Chironomidae metacommunities in stream systems of Brazil (Maxwell et al. 2021) and the Amazon Basin (Nicacio and Juen 2018) and are likely to be increasingly noted during periods of synchronous emergence of dominant taxa (Milošević et al. 2022).

We employed two spatial eigenvector mapping methods (i.e., dbMEM and AEM) to model aquatic and terrestrial dispersal pathways among chironomid communities. AEM exhibited higher explanatory power than dbMEM, suggesting that watercourse dispersal may play a more prominent role in structuring aquatic communities (Chiu et al. 2021; Padial et al. 2014). Our finding aligns with Nicacio and Juen (2018), who reported similar patterns in Chironomidae metacommunities. One possible explanation is that the overland dispersal of adult Chironomidae represents the briefest phase of the Chironomidae life cycle. Nonetheless, our data support the presence of overland dispersal, as adults of some typically freshwater species were recorded in estuarine sites, while the marine species *Polypedilum harteni* was detected at upstream freshwater sites (TCR‐M, YSOR‐U, and YSOR‐M), albeit at low abundances. These results highlight the intricate dispersal dynamics in stream chironomids, where both aquatic and terrestrial pathways contribute to community assembly (Robinson et al. 2002).

### 4.3 The Interplay of Dispersal and Ecological Tolerance in Shaping Chironomidae Biogeography

Our DNA barcode comparisons reveal that many Chironomidae species recorded in Hong Kong exhibit remarkably broad geographic and climatic distributions (Figures 7 and 8). This pattern appears counterintuitive, given that chironomids are often considered poor overland dispersers, with lateral movement of adults rarely exceeding 500 m (Delettre and Morvan 2000). However, previous studies likely underestimated their dispersal potential, as the ground‐level sampling methods employed fail to capture high‐altitude, wind‐assisted flight. Recent observations have documented chironomids at altitudes up to 160 m above ground level, indicating that adults can disperse passively over long distances via aerial currents (Atieli et al. 2023). Molecular evidence also supports the presence of wind‐assisted transoceanic dispersal, as reflected in the discordance between phylogenetic relationships and the geological sequence of Gondwana's breakup (Krosch et al. 2011b). In our dataset, the concentration of matches in monsoon‐influenced regions (Figure 7B) likely reflects how prevailing winds facilitate gene flow within metapopulations (Hu et al. 2025). Additional passive dispersal mechanisms may also contribute to broad distributions, including transport by migratory birds (Green et al. 2002) and human‐mediated dispersal (Gippet et al. 2019). Critically, such range expansions do not occur in single leaps but through multi‐generational “relay” colonization, where newly established populations serve as sources for subsequent dispersal events (Stefanescu et al. 2013; Hu et al. 2025).

Yet dispersal alone is insufficient, as successful population establishment requires compatibility with local climatic conditions. Temperature is widely recognized as a key driver of chironomid distributions, acting through both direct physiological effects and indirect habitat modifications (Eggermont and Heiri 2012; Lencioni and Rossaro 2005). Nevertheless, many chironomid species can complete larval development under suboptimal temperatures (Muñiz‐González et al. 2023; Foucault et al. 2018). For example, McKie et al. (2005) found that most observed chironomid species, including cool‐adapted Gondwanan taxa, occurred across the entire 20–1400 m elevational gradient in the Australian Wet Tropics, despite substantial temperature variation. At broader spatial scales, however, thermal constraints become evident because only a few matched species were recorded from temperate regions (Table 1), likely reflecting the reduced fitness of warm‐adapted species in cooler climates (Mackey 1977).

Compared to temperature, precipitation appears to impose a stronger constraint on Chironomidae distributions (Figure S4), as evidenced by the scarcity of species recorded in arid and semi‐arid regions of western and central Asia (Figure 7B). This pattern arises because chironomid species lacking drought‐resistance strategies rarely persist in streams prone to drying events (Canedo‐Argüelles et al. 2016). The extensive arid belt likely acts as a geographic barrier to dispersal and gene flow, explaining the very limited number of shared “molecular species” between Hong Kong and Europe (Figure S6).

These distributional patterns challenge traditional zoogeographic frameworks derived from terrestrial vertebrates. Classic boundaries between the Palaearctic and Oriental realms, such as the Yangtze River or the Qinling Mountains–Huai River line (Wallace 1876; Holt et al. 2013), do not act as effective barriers for freshwater insects like Chironomidae (Sæther 2000). Instead, our findings partially align with Bănărescu's (1992) Sino‐Indian Region for aquatic fauna, which encompasses East Asia and the Indo‐Malaysian subregion but excludes High Asia. Notably, Hong Kong marks the southern limit for several northward‐distributed species (Figure 8G), a pattern likely facilitated by the region's pronounced seasonal temperature fluctuations and mountainous terrain, which provide suitable habitats for cool‐adapted taxa (Dudgeon and Corlett 2011). Collectively, these results highlight a fundamental mismatch between terrestrial and freshwater biogeography, underscoring that conservation strategies based on vertebrate distribution patterns are insufficient to safeguard aquatic biodiversity (Abell et al. 2008).

### 4.4 Caveats and Future Perspectives

Hong Kong Chironomid species show divergent environmental preferences, leading to pronounced species replacement among habitats (Butakka et al. 2014). A study in Singapore found that only 8 of 314 species were shared between swamp forests and adjacent reservoirs (Baloğlu et al. 2018). Our study mainly focused on freshwater streams, and additional surveys across other habitats are needed to fully assess biodiversity (Rossaro and Marziali 2024; Přidalová et al. 2024). Further investigation should encompass a broader range of habitats in Hong Kong, such as wetlands, reservoirs, coastal zones, urban areas, and agricultural landscapes, to enhance understanding of the regional diversity and distribution of Chironomidae.

Although a large proportion of unexplained variation is typical in community ecological studies, the low explanatory power of our models (< 16%) may be partly attributable to limitations in study design and methodological constraints. First, limited spatial coverage of sampling sites resulted in a constrained environmental gradient, likely leading to an underestimation of environmental filtering effects. Furthermore, the absence of key biotic factors (e.g., fish biomass and aquatic vegetation cover), as well as the influence of historical contingency, partially contributes to the large proportion of unexplained variation, as both are recognized as important drivers of chironomid community structure (Milošević et al. 2022; Zheng et al. 2022). Additionally, the use of presence–absence data in this study omits ecological information derived from species abundance variation, which may further obscure the relationship between community dissimilarity and environmental predictors (Strayer 1999).

This study represents the first attempt at fauna comparison using publicly available DNA barcode data to investigate broad‐scale distribution patterns of Chironomidae. However, we admit that MOTUs do not align with the biological species concept (Collins and Cruickshank 2013; Taylor and Harris 2012), so species distributions derived from DNA barcoding may differ from the “true species” distributions. Additionally, the incomplete geographic and taxonomic coverage of public databases undermines the robustness of our findings as significant cryptic diversity remains unresolved (Han et al. 2023). In our study, 34.4% of newly generated barcodes failed to match public sequences at the species level using a 0.97 similarity threshold, underscoring significant gaps in reference libraries, which this study was designed to help fill. Furthermore, geographic biases potentially compromise the robustness of cross‐database barcode matching analysis, even though no direct correlation was found between regional barcode availability and sampling in this study. Despite these challenges, our findings highlight the substantial potential of barcode data for ecological and biogeographic research. Traditional faunal comparisons rely on high‐quality taxonomic data derived from morphological identification (Zhang and Corlett 2003), which is exceedingly difficult to obtain for taxonomically complex groups such as Chironomidae. In contrast, by leveraging substantial molecular data, such comparisons can be conducted efficiently at high taxonomic resolution and across global scales.

To address current limitations, future research should prioritize the development of more comprehensive reference libraries through international collaboration, with a particular focus on under‐sampled regions such as Africa, South America, and Central and Western Asia. We strongly recommend that detailed geographic and ecological metadata be included when submitting DNA barcode data to public repositories, as such information is essential for elucidating global species diversity patterns and for enhancing the utility of DNA barcodes in biodiversity assessment and conservation planning (Pentinsaari et al. 2020; Ratnasingham and Hebert 2007).

## 5 Conclusion

This study provides the first integrated assessment of Chironomidae diversity in Hong Kong by combining morphological and molecular approaches. The comprehensive barcode library generated serves as a valuable foundation for taxonomic research and metabarcoding‐based ecological applications. Environmental filtering and mass effects play important roles in structuring the Chironomidae community in Hong Kong, whereas the influence of dispersal limitation appears limited. The high diversity of Chironomidae and significant species turnover along subtle environmental gradients underscore their value as bioindicators in urban ecosystems. Cross‐database barcode matching analysis reveals that some chironomid species have broad geographic distributions, likely facilitated by wind‐mediated passive dispersal and wide ecological tolerance. By integrating taxonomic, ecological, and biogeographic analyses, our study provides an effective framework for understanding regional diversity patterns and their connectivity with surrounding regions.

## Author Contributions

**Wu Han:** conceptualization (equal), data curation (equal), formal analysis (equal), investigation (equal), methodology (equal), visualization (equal), writing – original draft (equal), writing – review and editing (equal). **Tsz‐Ying Chan:** conceptualization (equal), data curation (equal), investigation (equal), writing – review and editing (equal). **Chu‐Ming Zhang:** data curation (equal), writing – review and editing (equal). **Xiao‐Long Lin:** data curation (equal), writing – review and editing (equal). **Peter S. Cranston:** writing – review and editing (equal). **Thilina S. Nimalrathna:** writing – review and editing (equal). **Bai‐An Lin:** data curation (equal), writing – review and editing (equal). **Hong‐Qu Tang:** data curation (equal), writing – review and editing (equal). **Mathew Seymour:** conceptualization (equal), funding acquisition (lead), methodology (equal), project administration (equal), resources (equal), supervision (equal), writing – original draft (equal).

## Funding

This work was supported by the Environment and Conservation Fund (2022‐106).

## Conflicts of Interest

The authors declare no conflicts of interest.

## Supporting information


**Data S1:** ece373110‐sup‐0001‐supinfo.docx.

## Data Availability

All DNA barcode sequences generated in this study, along with their associated taxonomic data and geographic data, have been deposited in the public dataset DS‐CHIOHK on the BOLD database (DOI: https://doi.org/10.5883/DS‐CHIOHK).

## References

[ece373110-bib-0001] Abell, R. , M. L. Thieme , C. Revenga , et al. 2008. “Freshwater Ecoregions of the World: A New Map of Biogeographic Units for Freshwater Biodiversity Conservation.” Bioscience 58, no. 5: 403–414. 10.1641/B580507.

[ece373110-bib-0003] Andersen, T. , P. S. Cranston , and J. H. Epler . 2013. “The Larvae of Chironomidae (Diptera) of the Holarctic Region—Keys and Diagnoses.” Insect Systematics & Evolution 66: 1–556.

[ece373110-bib-0004] Armitage, P. D. , L. C. Pinder , and P. S. Cranston . 1995. The Chironomidae: Biology and Ecology of Non‐Biting Midges. Springer.

[ece373110-bib-0005] Astorga, A. , J. Heino , M. Luoto , and T. Muotka . 2011. “Freshwater Biodiversity at Regional Extent: Determinants of Macroinvertebrate Taxonomic Richness in Headwater Streams.” Ecography 34, no. 5: 705–713. 10.1111/j.1600-0587.2010.06427.x.

[ece373110-bib-0006] Atieli, H. E. , G. Zhou , D. Zhong , et al. 2023. “Wind‐Assisted High‐Altitude Dispersal of Mosquitoes and Other Insects in East Africa.” Journal of Medical Entomology 60, no. 4: 698–707. 10.1093/jme/tjad033. 37094808 PMC10337859

[ece373110-bib-0007] Baloğlu, B. , E. Clews , and R. Meier . 2018. “NGS Barcoding Reveals High Resistance of a Hyperdiverse Chironomid (Diptera) Swamp Fauna Against Invasion From Adjacent Freshwater Reservoirs.” Frontiers in Zoology 15, no. 1: 31. 10.1186/s12983-018-0276-7. 30127839 PMC6092845

[ece373110-bib-0009] Bănărescu, P. 1992. Zoogeography of Fresh Waters: Volume 2. Distribution and Dispersal of Freshwater Animals in North America and Eurasia, 519–1091. AULA‐Verlag.

[ece373110-bib-0011] Baselga, A. , and C. D. L. Orme . 2012. “Betapart: An R Package for the Study of Beta Diversity.” Methods in Ecology and Evolution 3, no. 5: 808–812. 10.1111/j.2041-210X.2012.00224.x.

[ece373110-bib-0012] Berg, M. B. , and R. A. Hellenthal . 1992. “The Role of Chironomidae in Energy Flow of a Lotic Ecosystem.” Netherlands Journal of Aquatic Ecology 26, no. 2: 471–476. 10.1007/BF02255277.

[ece373110-bib-0014] Blanchet, F. G. , P. Legendre , and D. Borcard . 2008a. “Modelling Directional Spatial Processes in Ecological Data.” Ecological Modelling 215, no. 4: 325–336. 10.1016/j.ecolmodel.2008.04.001.

[ece373110-bib-0015] Blanchet, F. G. , P. Legendre , and D. Borcard . 2008b. “Forward Selection of Explanatory Variables.” Ecology 89, no. 9: 2623–2632. 10.1890/07-0986.1. 18831183

[ece373110-bib-0016] Blattner, L. A. , P. Lapellegerie , C. Courtney‐Mustaphi , and O. Heiri . 2025. “Sediment Core DNA‐Metabarcoding and Chitinous Remain Identification: Integrating Complementary Methods to Characterise Chironomidae Biodiversity in Lake Sediment Archives.” Molecular Ecology Resources 25, no. 1: e14035. 10.1111/1755-0998.14035. 39434565 PMC11646301

[ece373110-bib-0017] Brown, B. L. , and C. M. Swan . 2010. “Dendritic Network Structure Constrains Metacommunity Properties in Riverine Ecosystems.” Journal of Animal Ecology 79, no. 3: 571–580. 10.1111/j.1365-2656.2010.01668.x. 20180874

[ece373110-bib-0018] Brown, S. , R. Collins , S. Boyer , et al. 2012. “SPIDER: An R Package for the Analysis of Species Identity and Evolution, With Particular Reference to DNA Barcoding.” Molecular Ecology Resources 12, no. 3: 562–565. 10.1111/j.1755-0998.2011.03108.x. 22243808

[ece373110-bib-0019] Buchner, D. , and F. Leese . 2020. “BOLDigger – A Python Package to Identify and Organise Sequences With the Barcode of Life Data Systems.” Metabarcoding and Metagenomics 4: e53535. 10.3897/mbmg.4.53535.

[ece373110-bib-0020] Butakka, C. M. M. , M. Grzybkowska , G. D. Pinha , and A. M. Takeda . 2014. “Habitats and Trophic Relationships of Chironomidae Insect Larvae From the Sepotuba River Basin, Pantanal of Mato Grosso, Brazil.” Brazilian Journal of Biology 74, no. 2: 395–407. 10.1590/1519-6984.26612. 25166324

[ece373110-bib-0022] Canedo‐Argüelles, M. , M. T. Bogan , D. A. Lytle , and N. Prat . 2016. “Are Chironomidae (Diptera) Good Indicators of Water Scarcity? Dryland Streams as a Case Study.” Ecological Indicators 71: 155–162. 10.1016/j.ecolind.2016.07.002.

[ece373110-bib-0023] Chase, J. M. , A. Jeliazkov , E. Ladouceur , and D. S. Viana . 2020. “Biodiversity Conservation Through the Lens of Metacommunity Ecology.” Annals of the New York Academy of Sciences 1469, no. 1: 86–104. 10.1111/nyas.14378. 32406120

[ece373110-bib-0024] Chimeno, C. , B. Rulik , A. Manfrin , G. Kalinkat , F. Hölker , and V. Baranov . 2023. “Facing the Infinity: Tackling Large Samples of Challenging Chironomidae (Diptera) With an Integrative Approach.” PeerJ 11: e15336. 10.7717/peerj.15336. 37250705 PMC10211366

[ece373110-bib-0025] Chiu, M. C. , S. Ao , V. H. Resh , F. He , and Q. Cai . 2021. “Species Dispersal Along Rivers and Streams May Have Variable Importance to Metapopulation Structure.” Science of the Total Environment 760: 144045. 10.1016/j.scitotenv.2020.144045. 33341625

[ece373110-bib-0026] Collins, R. A. , and R. H. Cruickshank . 2013. “The Seven Deadly Sins of DNA Barcoding.” Molecular Ecology Resources 13, no. 6: 969–975. 10.1111/1755-0998.12046. 23280099

[ece373110-bib-0027] Cowie, R. H. , P. Bouchet , and B. Fontaine . 2022. “The Sixth Mass Extinction: Fact, Fiction or Speculation?” Biological Reviews 97, no. 2: 640–663. 10.1111/brv.12816. 35014169 PMC9786292

[ece373110-bib-0028] Cranston, P. S. , and H. Tang . 2024. “An Identification Guide to the Genera of Aquatic Larval Chironomidae (Diptera) of South‐East Asia.” Zootaxa 5497, no. 2: 151–193. 39647157 10.11646/zootaxa.5497.2.1

[ece373110-bib-0030] Delettre, Y. R. , and N. Morvan . 2000. “Dispersal of Adult Aquatic Chironomidae (Diptera) in Agricultural Landscapes.” Freshwater Biology 44, no. 3: 399–411. 10.1046/j.1365-2427.2000.00578.x.

[ece373110-bib-0031] Dormann, C. F. , J. Elith , S. Bacher , et al. 2013. “Collinearity: A Review of Methods to Deal With It and a Simulation Study Evaluating Their Performance.” Ecography 36, no. 1: 27–46. 10.1111/j.1600-0587.2012.07348.x.

[ece373110-bib-0032] Dray, S. , D. Bauman , and G. Blanchet . 2025. “Adespatial: Multivariate Multiscale Spatial Analysis (Version 0.3‐28) [R Package].” https://CRAN.R‐project.org/package=adespatial.

[ece373110-bib-0033] Duan, H. , X. Lu , and G. Talamini . 2022. “The Impacts of Environmentally Mitigated River Channelization on Agriculture in Hong Kong.” Urbanie and Urbanus Journal 7: 145–156.

[ece373110-bib-0034] Dudgeon, D. , and R. T. Corlett . 2011. The Ecology and Biodiversity of Hong Kong. Cosmos Books & Lions Nature Education Foundation.

[ece373110-bib-0035] Dudgeon, D. , and D. L. Strayer . 2025. “Bending the Curve of Global Freshwater Biodiversity Loss: What Are the Prospects?” Biological Reviews 100, no. 1: 205–226. 39221642 10.1111/brv.13137 PMC11718631

[ece373110-bib-0036] Duffus, N. E. , A. Echeverri , L. Dempewolf , J. A. Noriega , P. R. Furumo , and J. Morimoto . 2023. “The Present and Future of Insect Biodiversity Conservation in the Neotropics: Policy Gaps and Recommendations.” Neotropical Entomology 52, no. 3: 407–421. 10.1007/s13744-023-01031-7. 36918492 PMC10181979

[ece373110-bib-0138] Edgar, R. C. 2004. “MUSCLE: A Multiple Sequence Alignment Method With Reduced Time and Space Complexity.” BMC Bioinformatics 5, no. 1: 113. 15318951 10.1186/1471-2105-5-113 PMC517706

[ece373110-bib-0037] Eggermont, H. , and O. Heiri . 2012. “The Chironomid–Temperature Relationship: Expression in Nature and Palaeoenvironmental Implications.” Biological Reviews 87, no. 2: 430–456. 10.1111/j.1469-185X.2011.00206.x. 22032243

[ece373110-bib-0038] Eisenhauer, N. , and J. Hines . 2021. “Invertebrate Biodiversity and Conservation.” Current Biology 31, no. 19: R1214–R1218. 10.1016/j.cub.2021.08.028. 34637734

[ece373110-bib-0039] Ekrem, T. 2002. “A Review of Selected South‐ and East Asian *Tanytarsus* vd Wulp (Diptera: Chironomidae).” Hydrobiologia 474, no. 1: 1–39. 10.1023/A:1016527603086.

[ece373110-bib-0040] Feio, M. J. , R. M. Hughes , S. R. Q. Serra , et al. 2023. “Fish and Macroinvertebrate Assemblages Reveal Extensive Degradation of the World's Rivers.” Global Change Biology 29, no. 2: 355–374. 10.1111/gcb.16439. 36131677 PMC10091732

[ece373110-bib-0041] Fick, S. E. , and R. J. Hijmans . 2017. “WorldClim 2: New 1‐Km Spatial Resolution Climate Surfaces for Global Land Areas.” International Journal of Climatology 37, no. 12: 4302–4315. 10.1002/joc.5086.

[ece373110-bib-0042] Folmer, O. , M. Black , W. Hoeh , R. Lutz , and R. Vrijenhoek . 1994. “DNA Primers for Amplification of Mitochondrial Cytochrome c Oxidase Subunit I From Diverse Metazoan Invertebrates.” Molecular Marine Biology and Biotechnology 3, no. 5: 294–299. 7881515

[ece373110-bib-0043] Fontes, J. T. , P. E. Vieira , T. Ekrem , P. Soares , and F. O. Costa . 2021. “BAGS: An Automated Barcode, Audit & Grade System for DNA Barcode Reference Libraries.” Molecular Ecology Resources 21, no. 3: 573–583. 10.1111/1755-0998.13262. 33000878

[ece373110-bib-0044] Foucault, Q. , A. Wieser , A. M. Waldvogel , B. Feldmeyer , and M. Pfenninger . 2018. “Rapid Adaptation to High Temperatures in *Chironomus riparius* .” Ecology and Evolution 8, no. 24: 12780–12789. 10.1002/ece3.4706. 30619582 PMC6308882

[ece373110-bib-0046] Gadawski, P. , M. Montagna , B. Rossaro , et al. 2022. “DNA Barcoding of Chironomidae From the Lake Skadar Region: Reference Library and a Comparative Analysis of the European Fauna.” Diversity and Distributions 28, no. 12: 2838–2857. 10.1111/ddi.13504.

[ece373110-bib-0047] Gippet, J. M. , A. M. Liebhold , G. Fenn‐Moltu , and C. Bertelsmeier . 2019. “Human‐Mediated Dispersal in Insects.” Current Opinion in Insect Science 35: 96–102. 10.1016/j.cois.2019.07.005. 31479895

[ece373110-bib-0048] Gomes, P. I. , and O. W. H. Wai . 2020. “Concrete Lined Urban Streams and Macroinvertebrates: A Hong Kong Case Study.” Urban Ecosystems 23, no. 1: 133–145. 10.1007/s11252-019-00898-y.

[ece373110-bib-0049] Gong, P. , H. Liu , M. N. Zhang , et al. 2019. “Stable Classification With Limited Sample: Transferring a 30‐m Resolution Sample Set Collected in 2015 to Mapping 10‐m Resolution Global Land Cover in 2017.” Science Bulletin 64, no. 6: 370–373. 10.1016/j.scib.2019.03.002. 36659725

[ece373110-bib-0050] Green, A. J. , J. Figuerola , and M. I. Sánchez . 2002. “Implications of Waterbird Ecology for the Dispersal of Aquatic Organisms.” Acta Oecologica 23, no. 3: 177–189. 10.1016/S1146-609X(02)01149-9.

[ece373110-bib-0051] Grönroos, M. , J. Heino , T. Siqueira , V. L. Landeiro , J. Kotanen , and L. M. Bini . 2013. “Metacommunity Structuring in Stream Networks: Roles of Dispersal Mode, Distance Type and Regional Environmental Context.” Ecology and Evolution 3, no. 13: 4473–4487. 10.1002/ece3.834. 24340188 PMC3856747

[ece373110-bib-0052] Han, W. , H. Tang , and Z. Ni . 2021. “DNA Barcodes and Morphology Reveal Two New Species of *Monodiamesa* Kieffer (Diptera: Chironomidae: Prodiamesinae) in Tibetan Plateau.” Zootaxa 4990, no. 1: 81–103. 10.11646/zootaxa.4990.1.5. 34186772

[ece373110-bib-0053] Han, W. , H. Tang , L. Wei , and E. Zhang . 2023. “The First DNA Barcode Library of Chironomidae From the Tibetan Plateau With an Evaluation of the Status of the Public Databases.” Ecology and Evolution 13, no. 2: e9849. 10.1002/ece3.9849. 36861023 PMC9969238

[ece373110-bib-0054] Han, W. , J. Wei , X. Lin , and H. Tang . 2020. “The Afro–Oriental Genus *Yaeprimus* Sasa et Suzuki (Diptera: Chironomidae: Chironomini): Phylogeny, New Species and Expanded Diagnoses.” Diversity 12, no. 1: 31. 10.3390/d12010031.

[ece373110-bib-0055] He, S. , J. Soininen , G. Deng , and B. Wang . 2020. “Metacommunity Structure of Stream Insects Across Three Hierarchical Spatial Scales.” Ecology and Evolution 10, no. 6: 2874–2884. 10.1002/ece3.6103. 32211162 PMC7083666

[ece373110-bib-0056] Hebert, P. D. , A. Cywinska , S. L. Ball , and J. R. DeWaard . 2003. “Biological Identifications Through DNA Barcodes.” Proceedings of the Royal Society of London. Series B: Biological Sciences 270, no. 1512: 313–321. 10.1098/rspb.2002.2218. PMC1691236 12614582

[ece373110-bib-0058] Heino, J. , and K. Korsu . 2008. “Testing Species‐Stone Area and Species‐Bryophyte Cover Relationships in Riverine Macroinvertebrates at Small Scales.” Freshwater Biology 53, no. 3: 558–568. 10.1111/j.1365-2427.2007.01920.x.

[ece373110-bib-0059] Heino, J. , A. S. Melo , T. Siqueira , J. Soininen , S. Valanko , and L. M. Bini . 2015. “Metacommunity Organisation, Spatial Extent and Dispersal in Aquatic Systems: Patterns, Processes and Prospects.” Freshwater Biology 60, no. 5: 845–869. 10.1111/fwb.12533.

[ece373110-bib-0060] Heino, J. , and H. Mykrä . 2008. “Control of Stream Insect Assemblages: Roles of Spatial Configuration and Local Environmental Factors.” Ecological Entomology 33, no. 5: 614–622. 10.1111/j.1365-2311.2008.01012.x.

[ece373110-bib-0061] Heino, J. , H. Mykrä , H. Hämäläinen , J. Aroviita , and T. Muotka . 2007. “Responses of Taxonomic Distinctness and Species Diversity Indices to Anthropogenic Impacts and Natural Environmental Gradients in Stream Macroinvertebrates.” Freshwater Biology 52, no. 9: 1846–1861. 10.1111/j.1365-2427.2007.01801.x.

[ece373110-bib-0062] Holt, B. G. , J. P. Lessard , M. K. Borregaard , et al. 2013. “An Update of Wallace's Zoogeographic Regions of the World.” Science 339, no. 6115: 74–78. 10.1126/science.1228282. 23258408

[ece373110-bib-0063] Hu, G. , H. Feng , A. Otuka , D. R. Reynolds , V. A. Drake , and J. W. Chapman . 2025. “The East Asian Insect Flyway: Geographical and Climatic Factors Driving Migration Among Diverse Crop Pests.” Annual Review of Entomology 70: 1–22. 10.1146/annurev-ento-012524-124018. 39499909

[ece373110-bib-0064] Kirk‐Spriggs, A. H. , and B. J. Sinclair . 2017. “Chironomidae.” In Manual of Afrotropical Diptera: Volume 2. Nematocerous Diptera and Lower Brachycera, edited by A. H. Kirk‐Spriggs and B. J. Sinclair , 813–863. South African National Biodiversity Institute.

[ece373110-bib-0065] Kranzfelder, P. , A. M. Anderson , A. T. Egan , et al. 2015. “Use of Chironomidae (Diptera) Surface‐Floating Pupal Exuviae as a Rapid Bioassessment Protocol for Water Bodies.” Journal of Visualized Experiments 101: e52558. 10.3791/52558. PMC4545202 26274889

[ece373110-bib-0066] Krosch, M. , and P. S. Cranston . 2012. “Non‐Destructive DNA Extraction From Chironomidae, Including of Fragile Pupal Exuviae, Extends Analysable Collections and Enhances Vouchering.” CHIRONOMUS Journal of Chironomidae Research 25: 22–27. 10.5324/cjcr.v0i25.1532.

[ece373110-bib-0067] Krosch, M. N. , A. M. Baker , P. B. Mather , and P. S. Cranston . 2011a. “Spatial Population Genetic Structure Reveals Strong Natal Site Fidelity in *Echinocladius* Martini (Diptera: Chironomidae) in Northeast Queensland, Australia.” Freshwater Biology 56, no. 7: 1328–1341.

[ece373110-bib-0068] Krosch, M. N. , A. M. Baker , P. B. Mather , and P. S. Cranston . 2011b. “Systematics and Biogeography of the Gondwanan Orthocladiinae (Diptera: Chironomidae).” Molecular Phylogenetics and Evolution 59, no. 2: 458–468. 10.1016/j.ympev.2011.03.003. 21402162

[ece373110-bib-0069] Lai, J. , Y. Zou , J. Zhang , and P. R. Peres‐Neto . 2022. “Generalizing Hierarchical and Variation Partitioning in Multiple Regression and Canonical Analyses Using the Rdacca.Hp R Package.” Methods in Ecology and Evolution 13, no. 4: 782–788. 10.1111/2041-210X.13800.

[ece373110-bib-0070] Lau, C. S. K. 2025. Checklist of Insects of Hong Kong. 2nd ed, 334–335. Agriculture, Fisheries and Conservation Department.

[ece373110-bib-0071] Legendre, P. , and O. Gauthier . 2014. “Statistical Methods for Temporal and Space–Time Analysis of Community Composition Data.” Proceedings of the Royal Society B: Biological Sciences 281, no. 1778: 20132728. 10.1098/rspb.2013.2728. PMC3906937 24430848

[ece373110-bib-0072] Leibold, M. A. , M. Holyoak , N. Mouquet , et al. 2004. “The Metacommunity Concept: A Framework for Multi‐Scale Community Ecology.” Ecology Letters 7, no. 7: 601–613. 10.1111/j.1461-0248.2004.00608.x.

[ece373110-bib-0073] Lencioni, V. , P. Cranston , and E. Makarchenko . 2018. “Recent Advances in the Study of Chironomidae: An Overview.” Journal of Limnology 77, no. 1: 1–6. 10.4081/jlimnol.2018.1865.

[ece373110-bib-0074] Lencioni, V. , and B. Rossaro . 2005. “Microdistribution of Chironomids (Diptera: Chironomidae) in Alpine Streams: An Autoecological Perspective.” Hydrobiologia 533, no. 1: 61–76. 10.1007/s10750-004-2393-x.

[ece373110-bib-0075] Leszczyńska, J. , M. Grzybkowska , Ł. Głowacki , and M. Dukowska . 2019. “Environmental Variables Influencing Chironomid Assemblages (Diptera: Chironomidae) in Lowland Rivers of Central Poland.” Environmental Entomology 48, no. 4: 988–997. 10.1093/ee/nvz057. 31157378

[ece373110-bib-0076] Ligeiro, R. , R. M. Hughes , P. R. Kaufmann , et al. 2013. “Defining Quantitative Stream Disturbance Gradients and the Additive Role of Habitat Variation to Explain Macroinvertebrate Taxa Richness.” Ecological Indicators 25: 45–57. 10.1016/j.ecolind.2012.09.004.

[ece373110-bib-0077] Lin, X. L. , E. Stur , and T. Ekrem . 2018. “DNA Barcodes and Morphology Reveal Unrecognized Species in Chironomidae (Diptera).” Insect Systematics & Evolution 49, no. 4: 329–398. 10.1163/1876312X-00002184.

[ece373110-bib-0078] Liu, W. B. , Y. Yao , T. Chang , C. C. Yan , and X. L. Lin . 2022. “Contribution to the Knowledge of *Rheotanytarsus pellucidus* Species Group From China (Diptera, Chironomidae): Three New and One Newly Recorded Species.” Zootaxa 5188, no. 2: 145–156. 10.11646/zootaxa.5188.2.4. 37044788

[ece373110-bib-0079] Mace, G. M. , K. Norris , and A. H. Fitter . 2012. “Biodiversity and Ecosystem Services: A Multilayered Relationship.” Trends in Ecology & Evolution 27, no. 1: 19–26. 10.1016/j.tree.2011.08.006. 21943703

[ece373110-bib-0080] Mackey, A. P. 1977. “Growth and Development of Larval Chironomidae.” Oikos 28, no. 2–3: 270–275. 10.2307/3543981.

[ece373110-bib-0081] Makarchenko, E. A. , A. A. Semenchenko , and D. M. Palatov . 2023. “Fauna and Taxonomy of *Diamesinae* (Diptera, Chironomidae) From the Caucasus, With a Morphological Description and DNA Barcoding of New Taxa and a Discussion of Diagnostic Problems for *Diamesa* Meigen and *Pseudodiamesa* Goetghebuer.” Zootaxa 5271, no. 2: 313–328. 10.11646/zootaxa.5271.2.6. 37518126

[ece373110-bib-0082] Matthews‐Bird, F. , W. D. Gosling , A. L. Coe , et al. 2016. “Environmental Controls on the Distribution and Diversity of Lentic Chironomidae (Insecta: Diptera) Across an Altitudinal Gradient in Tropical South America.” Ecology and Evolution 6, no. 1: 91–112. 10.1002/ece3.1833. 26811777 PMC4716524

[ece373110-bib-0083] Maxwell, M. F. , E. Secretti , M. M. Pires , and C. B. Kotzian . 2021. “The Relative Importance of Spatial and Environmental Processes in the Assembly of Larval Chironomidae (Insecta, Diptera) Communities Along a Transition Landscape in Southern Brazilian Streams.” Limnology 22, no. 2: 259–268. 10.1007/s10201-021-00652-4.

[ece373110-bib-0084] McKie, B. G. , R. G. Pearson , and P. S. Cranston . 2005. “Does Biogeographical History Matter? Diversity and Distribution of Lotic Midges (Diptera: Chironomidae) in the Australian Wet Tropics.” Austral Ecology 30, no. 1: 69–81. 10.1111/j.1442-9993.2005.01408.x.

[ece373110-bib-0085] Milošević, D. , A. S. Medeiros , D. Cvijanović , et al. 2022. “Implications of Local Niche‐ and Dispersal‐Based Factors That May Influence Chironomid Assemblages in Bioassessment.” Environmental Science and Pollution Research 29, no. 34: 51951–51963. 10.1007/s11356-022-19302-y. 35257340

[ece373110-bib-0086] Muñiz‐González, A. B. , J. L. Martínez‐Guitarte , and V. Lencioni . 2023. “Impact of Global Warming on Kryal Fauna: Thermal Tolerance Response of *Diamesa steinboecki* (Goetghebuer, 1933; Chironomidae).” Diversity 15, no. 6: 708. 10.3390/d15060708.

[ece373110-bib-0087] Nicacio, G. , and L. Juen . 2015. “Chironomids as Indicators in Freshwater Ecosystems: An Assessment of the Literature.” Insect Conservation and Diversity 8, no. 5: 393–403. 10.1111/icad.12123.

[ece373110-bib-0088] Nicacio, G. , and L. Juen . 2018. “Relative Roles of Environmental and Spatial Constraints in Assemblages of Chironomidae (Diptera) in Amazonian Floodplain Streams.” Hydrobiologia 820, no. 1: 201–213. 10.1007/s10750-018-3657-1.

[ece373110-bib-0089] Niitsuma, H. 2013. “Revision of the Japanese *Ablabesmyia* (Diptera: Chironomidae: Tanypodinae), With Descriptions of Three New Species.” Zootaxa 3664, no. 4: 479–504. 10.11646/zootaxa.3664.4.4. 26266315

[ece373110-bib-0090] Oksanen, J. , F. G. Blanchet , M. Friendly , et al. 2022. “vegan: Community Ecology Package (Version 2.6–4) [R Package].” https://cran.r‐project.org/package=vegan.

[ece373110-bib-0091] Or, C. K. M. , and B. P. L. Chan . 2025. The State of Hong Kong Biodiversity 2025, 10. WWF‐Hong Kong.

[ece373110-bib-0092] Orendt, C. 1999. “Chironomids as Bioindicators in Acidified Streams: A Contribution to the Acidity Tolerance of Chironomid Species With a Classification in Sensitivity Classes.” International Review of Hydrobiology 84, no. 5: 439–449. 10.1002/iroh.199900038.

[ece373110-bib-0093] Padial, A. A. , F. Ceschin , S. A. Declerck , et al. 2014. “Dispersal Ability Determines the Role of Environmental, Spatial and Temporal Drivers of Metacommunity Structure.” PLoS One 9, no. 10: e111227. 10.1371/journal.pone.0111227. 25340577 PMC4207762

[ece373110-bib-0094] Paradis, E. , and K. Schliep . 2019. “Ape 5.0: An Environment for Modern Phylogenetics and Evolutionary Analyses in R.” Bioinformatics 35, no. 3: 526–528. 10.1093/bioinformatics/bty633. 30016406

[ece373110-bib-0095] Pentinsaari, M. , S. Ratnasingham , S. E. Miller , and P. D. Hebert . 2020. “BOLD and GenBank Revisited – Do Identification Errors Arise in the Lab or in the Sequence Libraries?” PLoS One 15, no. 4: e0231814. 10.1371/journal.pone.0231814. 32298363 PMC7162515

[ece373110-bib-0096] Pinder, L. C. V. 1986. “Biology of Freshwater Chironomidae.” Annual Review of Entomology 31, no. 1: 1–23. 10.1146/annurev.en.31.010186.000245.

[ece373110-bib-0097] Přidalová, M. S. , L. Hamerlík , M. Novikmec , et al. 2024. “Diversity and Distribution of Chironomids in Central European Ponds.” Ecology and Evolution 14, no. 5: e11354. 10.1002/ece3.11354. 38711486 PMC11070637

[ece373110-bib-0098] Puillandre, N. , S. Brouillet , and G. Achaz . 2021. “ASAP: Assemble Species by Automatic Partitioning.” Molecular Ecology Resources 21, no. 2: 609–620. 10.1111/1755-0998.13281. 33058550

[ece373110-bib-0099] R Core Team . 2025. R: A Language and Environment for Statistical Computing. R Foundation for Statistical Computing. https://R‐project.org.

[ece373110-bib-0100] Ratnasingham, S. , and P. D. Hebert . 2013. “A DNA‐Based Registry for All Animal Species: The Barcode Index Number (BIN) System.” PLoS One 8, no. 7: e66213. 10.1371/journal.pone.0066213. 23861743 PMC3704603

[ece373110-bib-0101] Ratnasingham, S. , and P. D. N. Hebert . 2007. “BOLD: The Barcode of Life Data System (http://www.barcodinglife.org).” Molecular Ecology Notes 7, no. 3: 355–364. 10.1111/j.1471-8286.2007.01678.x. 18784790 PMC1890991

[ece373110-bib-0102] Rico, E. , and A. Quesada . 2013. “Distribution and Ecology of Chironomids (Diptera, Chironomidae) on Byers Peninsula, Maritime Antarctica.” Antarctic Science 25, no. 2: 288–291. 10.1017/S095410201200096X.

[ece373110-bib-0103] Robinson, C. T. , K. Tockner , and J. V. Ward . 2002. “The Fauna of Dynamic Riverine Landscapes.” Freshwater Biology 47, no. 4: 661–677. 10.1046/j.1365-2427.2002.00921.x.

[ece373110-bib-0105] Rossaro, B. , and L. Marziali . 2024. “Response of Chironomids (Diptera, Chironomidae) to Environmental Factors at Different Spatial Scales.” Insects 15, no. 4: 272. 10.3390/insects15040272. 38667402 PMC11050053

[ece373110-bib-0106] Rossaro, B. , L. Marziali , and A. Boggero . 2022. “Response of Chironomids to Key Environmental Factors: Perspective for Biomonitoring.” Insects 13, no. 10: 911. 10.3390/insects13100911. 36292859 PMC9604178

[ece373110-bib-0107] Sæther, O. A. 1980. “A Glossary of Chironomid Morphology Terminology (Diptera: Chironomidae).” Entomologica Scandinavica Supplement 14: 1–51.

[ece373110-bib-0108] Sæther, O. A. 2000. “Zoogeographical Patterns in Chironomidae (Diptera).” SIL Proceedings, 1922‐2010 27, no. 1: 290–302. 10.1080/03680770.1998.11901242.

[ece373110-bib-0110] Šamulková, M. , Z. Čiamporová‐Zaťovičová , F. Čiampor , K. Tuhrinová , and P. Macko . 2025. “Is the DNA Barcode Database Fit for Purpose? Assessing the Feasibility of Reverse Taxonomy in Five Key Aquatic Insect Orders Relevant to Freshwater Biomonitoring Under the Water Framework Directive.” Biologia 80, no. 10: 1. 10.1007/s11756-025-01999-0.

[ece373110-bib-0111] Serra, S. R. Q. , M. A. S. Graça , S. Dolédec , and M. J. Feio . 2017. “Chironomidae Traits and Life History Strategies as Indicators of Anthropogenic Disturbance.” Environmental Monitoring and Assessment 189, no. 7: 326. 10.1007/s10661-017-6027-y. 28600682

[ece373110-bib-0113] Seymour, M. , K. Deiner , and F. Altermatt . 2016. “Scale and Scope Matter When Explaining Varying Patterns of Community Diversity in Riverine Metacommunities.” Basic and Applied Ecology 17, no. 2: 134–144. 10.1016/j.baae.2015.10.007.

[ece373110-bib-0114] Seymour, M. , F. K. Edwards , B. J. Cosby , et al. 2021. “Environmental DNA Provides Higher Resolution Assessment of Riverine Biodiversity and Ecosystem Function via Spatio‐Temporal Nestedness and Turnover Partitioning.” Communications Biology 4, no. 1: 512. 10.1038/s42003-021-02031-2. 33941836 PMC8093236

[ece373110-bib-0115] Seymour, M. , T. Roslin , J. R. deWaard , et al. 2024. “Global Arthropod Beta‐Diversity Is Spatially and Temporally Structured by Latitude.” Communications Biology 7: 552. 10.1038/s42003-024-06199-1. 38720028 PMC11078949

[ece373110-bib-0116] Song, C. , X. L. Lin , Q. Wang , and X. H. Wang . 2018. “DNA Barcodes Successfully Delimit Morphospecies in a Superdiverse Insect Genus.” Zoologica Scripta 47, no. 3: 311–324. 10.1111/zsc.12284.

[ece373110-bib-0117] Stefanescu, C. , F. Páramo , S. Åkesson , et al. 2013. “Multi‐Generational Long‐Distance Migration of Insects: Studying the Painted Lady Butterfly in the Western Palaearctic.” Ecography 36, no. 4: 474–486. 10.1111/j.1600-0587.2012.07738.x.

[ece373110-bib-0118] Strayer, D. L. 1999. “Statistical Power of Presence‐Absence Data to Detect Population Declines.” Conservation Biology 13, no. 5: 1034–1038. 10.1046/j.1523-1739.1999.98143.x.

[ece373110-bib-0119] Strayer, D. L. , and D. Dudgeon . 2010. “Freshwater Biodiversity Conservation: Recent Progress and Future Challenges.” Journal of the North American Benthological Society 29, no. 1: 344–358. 10.1899/08-171.1.

[ece373110-bib-0120] Stur, E. , and T. Ekrem . 2011. “Exploring Unknown Life Stages of Arctic Tanytarsini (Diptera: Chironomidae) With DNA Barcoding.” Zootaxa 2743, no. 1: 27–39. 10.11646/zootaxa.2743.1.2.

[ece373110-bib-0121] Taberlet, P. , E. Coissac , F. Pompanon , C. Brochmann , and E. Willerslev . 2012. “Towards Next‐Generation Biodiversity Assessment Using DNA Metabarcoding.” Molecular Ecology 21, no. 8: 2045–2050. 10.1111/j.1365-294X.2012.05470.x. 22486824

[ece373110-bib-0122] Tang, H. Q. , and H. Niitsuma . 2017. “Review of the Japanese *Microtendipes* (Diptera: Chironomidae: Chironominae), With Description of a New Species.” Zootaxa 4320, no. 3: 535–553. 10.11646/zootaxa.4320.3.8.

[ece373110-bib-0123] Taylor, H. R. , and W. E. Harris . 2012. “An Emergent Science on the Brink of Irrelevance: A Review of the Past 8 Years of DNA Barcoding.” Molecular Ecology Resources 12, no. 3: 377–388. 10.1111/j.1755-0998.2012.03119.x. 22356472

[ece373110-bib-0124] Thompson, P. L. , L. M. Guzman , L. De Meester , et al. 2020. “A Process‐Based Metacommunity Framework Linking Local and Regional Scale Community Ecology.” Ecology Letters 23, no. 9: 1314–1329. 10.1111/ele.13568. 32672410 PMC7496463

[ece373110-bib-0125] Tonkin, J. D. , F. Altermatt , D. S. Finn , et al. 2018. “The Role of Dispersal in River Network Metacommunity Frameworks: Patterns, Processes, and Pathways.” Freshwater Biology 63, no. 1: 141–163. 10.1111/fwb.13037.

[ece373110-bib-0126] Vermonden, K. , R. S. E. W. Leuven , G. der Van Velde , M. M. Van Katwijk , J. G. M. Roelofs , and A. J. Hendriks . 2009. “Urban Drainage Systems: An Undervalued Habitat for Aquatic Macroinvertebrates.” Biological Conservation 142, no. 5: 1105–1115. 10.1016/j.biocon.2009.01.026.

[ece373110-bib-0127] Wallace, A. R. 1876. The Geographical Distribution of Animals. Macmillan.

[ece373110-bib-0128] Wang, X. 1995. “ *Rheocricotopus (R.) orientalis* a New Species From China (Diptera: Chironomidae).” Aquatic Insects 17, no. 1: 37–40. 10.1080/01650429509361567.

[ece373110-bib-0129] Weiskopf, S. R. , B. J. E. Myers , M. I. Arce‐Plata , et al. 2022. “A Conceptual Framework to Integrate Biodiversity, Ecosystem Function, and Ecosystem Service Models.” Bioscience 72, no. 11: 1062–1073. 10.1093/biosci/biac074. 36506699 PMC9718641

[ece373110-bib-0130] Wickham, H. 2010. “A Layered Grammar of Graphics.” Journal of Computational and Graphical Statistics 19, no. 1: 3–28. 10.1198/jcgs.2009.07098.

[ece373110-bib-0131] Wiederholm, T. 1986. “Chironomidae of the Holarctic Region. Part 2. Pupae.” Entomologica Scandinavica Supplement 28: 1–482.

[ece373110-bib-0133] Xu, Y. , X. Xu , and Q. Tang . 2016. “Human Activity Intensity of Land Surface: Concept, Methods and Application in China.” Journal of Geographical Sciences 26, no. 9: 1349–1361. 10.1007/s11442-016-1331-y.

[ece373110-bib-0134] Zhang, L. , and R. T. Corlett . 2003. “Phytogeography of Hong Kong Bryophytes.” Journal of Biogeography 30, no. 9: 1329–1337. 10.1046/j.1365-2699.2003.00947.x.

[ece373110-bib-0135] Zhang, R. , C. Song , X. Qi , and X. Wang . 2016. “Taxonomic Review on the Subgenus *Tripodura* Townes (Diptera: Chironomidae: *Polypedilum*) From China With Eleven New Species and a Supplementary World Checklist.” Zootaxa 4136, no. 1: 1–53. 27395703 10.11646/zootaxa.4136.1.1

[ece373110-bib-0136] Zheng, W. , E. Zhang , R. Wang , and P. G. Langdon . 2022. “Human Impacts Alter Driver–Response Relationships in Lakes of Southwest China.” Limnology and Oceanography 67: S390–S402. 10.1002/lno.11946.

[ece373110-bib-0137] Zinchenko, T. D. , L. V. Golovatyuk , and E. V. Abrosimova . 2019. “Non‐Biting Midges (Diptera, Chironomidae) in the Benthic Communities of Saline Rivers in the Lake Elton Basin: Diversity, Salinity Tolerance, and Distribution.” Entomological Review 99, no. 6: 820–835. 10.1134/S0013873819060095.

