# Supplementary material for: Integrative Assessment of Hong Kong Chironomidae (Diptera) Shows High Species Richness Linked to Spatial and Environmental Factors
Source: Ecol Evol. 2026 Feb 15;16(2):e73110. doi: 10.1002/ece3.73110 (PMC12907583; doi:10.1002/ece3.73110)
Supplement: Supplementary file 1 — Data S1: ece373110‐sup‐0001‐supinfo.docx. [file ECE3-16-e73110-s001.docx]

**Supporting Information for:**

Integrative assessment of Hong Kong Chironomidae (Diptera) shows high species richness linked to spatial and environmental factors

Wu Han^1^, Tsz-Ying Chan^1^, Chu-Ming Zhang^1^, Xiao-Long Lin^2#^, Peter S. Cranston^3^, Thilina S. Nimalrathna^1^, Bai-an Lin^1^, Hong-Qu Tang^4ψ^, Mathew Seymour^1*^

1. School of Biological Sciences, The University of Hong Kong, Hong Kong SAR, China

2. Engineering Research Center of Environmental DNA and Ecological Water Health Assessment, Shanghai Ocean University, Shanghai 201306, China

3. Evolution & Ecology, Australian National University, Canberra, ACT 0200, Australia

4. Life Science and Technology College, Jinan University, Guangzhou 510632, China

Co-corresponding authors:

* mat.seymour@gmail.com (M. Seymour)

^#^ lin880224@gmail.com (X.-L. Lin)

^ψ^ townningt@gmail.com(H.Q. Tang)

**Supporting Information for:**

Integrative assessment of Hong Kong Chironomidae (Diptera) shows high species richness linked to spatial and environmental factors

**Supporting Information includes 3 tables and 7 figures:**

**Table S1.** Sampling site codes and geographic informations

**Table S2.** Generic-level checklist of Hong Kong Chironomidae (Diptera)

**Table S3.** Summary of beta diversity and its decomposed components

**Figure S1.** Partial Mantel correlations between biodiversity index and environmental variables

**Figure S2.** Biplots of Distance-based Redundancy Analysis

**Figure S3.** Climatic niche profiles of chironomid taxa matched with Hong Kong assemblages

**Figure S4.** Heat map of chironomid species richness across climatic conditions

**Figure S5.** Number of shared chironomid species across global regions

**Figure S6.** Geographic distribution pattern of the matched species (100% identity threshold)

**Figure S7.** Relationship between global barcode data availability and species matches with Hong Kong

**Tables**

**Table S1.** Sampling site codes and geographic information.

| **Code** | **Watershed** | **Section** | **Latitude (N°)** | **Longitude (E°)** | **Elevation (m)** |
| --- | --- | --- | --- | --- | --- |
| TCR-U | Tung Chung | upstream | 22.2689 | 113.9331 | 123 |
| TCR-M | Tung Chung | midstream | 22.2769 | 113.9297 | 29 |
| TCR-D | Tung Chung | downstream | 22.2805 | 113.9301 | 12 |
| LTR-U | Lam Tsuen | upstream | 22.4413 | 114.1301 | 299 |
| LTR-M | Lam Tsuen | midstream | 22.4609 | 114.1413 | 100 |
| LTR-D | Lam Tsuen | downstream | 22.4535 | 114.1530 | 37 |
| YSOR-U | Yung ShueO | upstream | 22.4255 | 114.2929 | 13 |
| YSOR-M | Yung ShueO | midstream | 22.4257 | 114.2908 | 49 |
| YSOR-D | Yung ShueO | downstream | 22.4270 | 114.2886 | 34 |
| MLHHR-U | Mai Lai Hau Hang | upstream | 22.3708 | 114.2119 | 353 |
| MLHHR-M | Mai Lai Hau Hang | midstream | 22.3746 | 114.2129 | 214 |
| MLHHR-D | Mai Lai Hau Hang | downstream | 22.3756 | 114.2120 | 136 |
| DWBR-U | Deep Water Bay | upstream | 22.2506 | 114.1860 | 84 |
| DWBR-M | Deep Water Bay | midstream | 22.2494 | 114.1879 | 53 |
| DWBR-D | Deep Water Bay | downstream | 22.2472 | 114.1872 | 20 |

**Table S2.** A checklist of Chironomidae (Diptera) at the generic level from five streams in Hong Kong, including the number of species per material type

| **Subfamily** | **Genus** | **Species: M** | **Species: P** | **Species: L** |
| --- | --- | --- | --- | --- |
| Chironominae | *Ainuyusurika* | 1 | 0 | 1 |
| Chironominae | *Chironomus* | 6 | 0 | 0 |
| Chironominae | *Cladopelma* | 1 | 0 | 0 |
| Chironominae | *Cladotanytarsus* | 2 | 0 | 0 |
| Chironominae | *Cryptochironomus* | 2 | 0 | 0 |
| Chironominae | *Cryptotendipes* | 1 | 0 | 0 |
| Chironominae | *Demicryptochironomus* | 4 | 0 | 0 |
| Chironominae | *Dicrotendipes* | 2 | 0 | 1 |
| Chironominae | *Harnischia* | 3 | 0 | 0 |
| Chironominae | *Kiefferulus* | 2 | 0 | 0 |
| Chironominae | *Kribiodosis* | 1 | 0 | 0 |
| Chironominae | *Microchironomus* | 1 | 0 | 0 |
| Chironominae | *Microtendipes* | 6 | 1 | 2 |
| Chironominae | *Neozavrelia* | 2 | 0 | 0 |
| Chironominae | *Nilothauma* | 4 | 0 | 0 |
| Chironominae | *Olecryptotendipes* | 1 | 0 | 0 |
| Chironominae | *Paratanytarsus* | 2 | 0 | 0 |
| Chironominae | *Paratendipes* | 1 | 0 | 0 |
| Chironominae | *Polypedilum* | 44 | 12 | 8 |
| Chironominae | *Rheotanytarsus* | 14 | 5 | 3 |
| Chironominae | *Stempellina* | 1 | 1 | 1 |
| Chironominae | *Stempellinella* | 0 | 1 | 1 |
| Chironominae | *Stenochironomus* | 3 | 0 | 1 |
| Chironominae | *Sublettea* | 1 | 1 | 0 |
| Chironominae | *Tanytarsus* | 16 | 5 | 5 |
| Chironominae | *Tribelos* | 1 | 0 | 0 |
| Chironominae | *Yaeprimus* | 2 | 0 | 0 |
| Chironominae | *Yaetanytarsus* | 2 | 0 | 0 |
| **Subfamily** | ***Genus*** | **Species: M** | **Species: P** | **Species: L** |
| Chironominae | *Zhouomyia* | 1 | 0 | 0 |
| Orthocladiinae | *Antillocladius* | 1 | 0 | 0 |
| Orthocladiinae | *Austrobrillia* | 1 | 0 | 1 |
| Orthocladiinae | *Bryophaenocladius* | 3 | 0 | 0 |
| Orthocladiinae | *Cardiocladius* | 1 | 0 | 0 |
| Orthocladiinae | *Compterosmittia* | 4 | 0 | 0 |
| Orthocladiinae | *Corynoneura* | 7 | 1 | 1 |
| Orthocladiinae | *Cricotopus* | 8 | 6 | 4 |
| Orthocladiinae | *Eukiefferiella* | 2 | 0 | 2 |
| Orthocladiinae | *Heleniella* | 1 | 0 | 0 |
| Orthocladiinae | *Krenosmittia* | 1 | 0 | 0 |
| Orthocladiinae | *Limnophyes* | 5 | 0 | 0 |
| Orthocladiinae | *Metriocnemus* | 0 | 0 | 0 |
| Orthocladiinae | *Nanocladius* | 1 | 1 | 0 |
| Orthocladiinae | *Parachaetocladius* | 2 | 0 | 0 |
| Orthocladiinae | *Paracricotopus* | 2 | 0 | 0 |
| Orthocladiinae | *Parakiefferiella* | 1 | 0 | 0 |
| Orthocladiinae | *Parametriocnemus* | 4 | 1 | 1 |
| Orthocladiinae | *Paraphaenocladius* | 2 | 1 | 1 |
| Orthocladiinae | *Psectrocladius* | 1 | 0 | 0 |
| Orthocladiinae | *Pseudorthocladius* | 5 | 0 | 0 |
| Orthocladiinae | *Pseudosmittia* | 14 | 0 | 1 |
| Orthocladiinae | *Rheocricotopus* | 2 | 1 | 1 |
| Orthocladiinae | *Smittia* | 2 | 0 | 0 |
| Orthocladiinae | *Thienemanniella* | 4 | 2 | 2 |
| Orthocladiinae | *Tvetenia* | 0 | 0 | 1 |
| Podonominae | *Paraboreochlus* | 1 | 0 | 0 |
| Tanypodinae | *Ablabesmyia* | 6 | 1 | 0 |
| Tanypodinae | *Coffmania* | 1 | 1 | 1 |
| Tanypodinae | *Conchapelopia* | 1 | 2 | 2 |
| **Subfamily** | ***Genus*** | **Species: M** | **Species: P** | **Species: L** |
| Tanypodinae | *Denopelopia* | 1 | 0 | 0 |
| Tanypodinae | *Larsia* | 1 | 0 | 1 |
| Tanypodinae | *Natarsia* | 1 | 0 | 0 |
| Tanypodinae | *Nilotanypus* | 1 | 1 | 1 |
| Tanypodinae | *Procladius* | 1 | 0 | 0 |
| Tanypodinae | *Thienemannimyia* | 2 | 0 | 0 |
| Tanypodinae | *Zavrelimyia* | 9 | 2 | 3 |

Note: M: adult male; P: pupa; L: larva.

**Table S3.** Partial Mantel test results assessing the association between chironomid community dissimilarity and individual environmental variables, while controlling for spatial autocorrelation.

| Variables | r | p |
| --- | --- | --- |
| WT (°C) | 0.679 | **0.001** |
| pH | 0.014 | 0.412 |
| Turbidity (NTU) | 0.069 | 0.275 |
| DO (mg/L) | 0.08 | 0.32 |
| Salinity (ppt) | 0.757 | **0.005** |
| Velocity (m/s) | 0.415 | **0.028** |
| Elevation (m) | 0.12 | 0.238 |
| Sand (%) | 0.77 | **0.003** |
| Gravel (%) | -0.098 | 0.684 |
| Cobble (%) | 0.174 | 0.16 |
| Boulder (%) | 0.362 | **0.002** |

Note: Variables reaching statistical significance (p < 0.05) are presented in bold.

**Table S4.** Summary of beta diversity and its decomposed components with comparison of within and between stream sites.

|  | Within Stream | | | | Between Stream | | | |
| --- | --- | --- | --- | --- | --- | --- | --- | --- |
|  | min | max | mean | sd | min | max | mean | sd |
| Turnover | 0.333 | 0.733 | 0.536 | 0.115 | 0.353 | 0.933 | 0.651 | 0.137 |
| Nestedness | 0.013 | 0.42 | 0.125 | 0.114 | 0.004 | 0.391 | 0.098 | 0.077 |
| Sørensen | 0.448 | 0.944 | 0.661 | 0.17 | 0.556 | 0.972 | 0.749 | 0.117 |

**Figures**


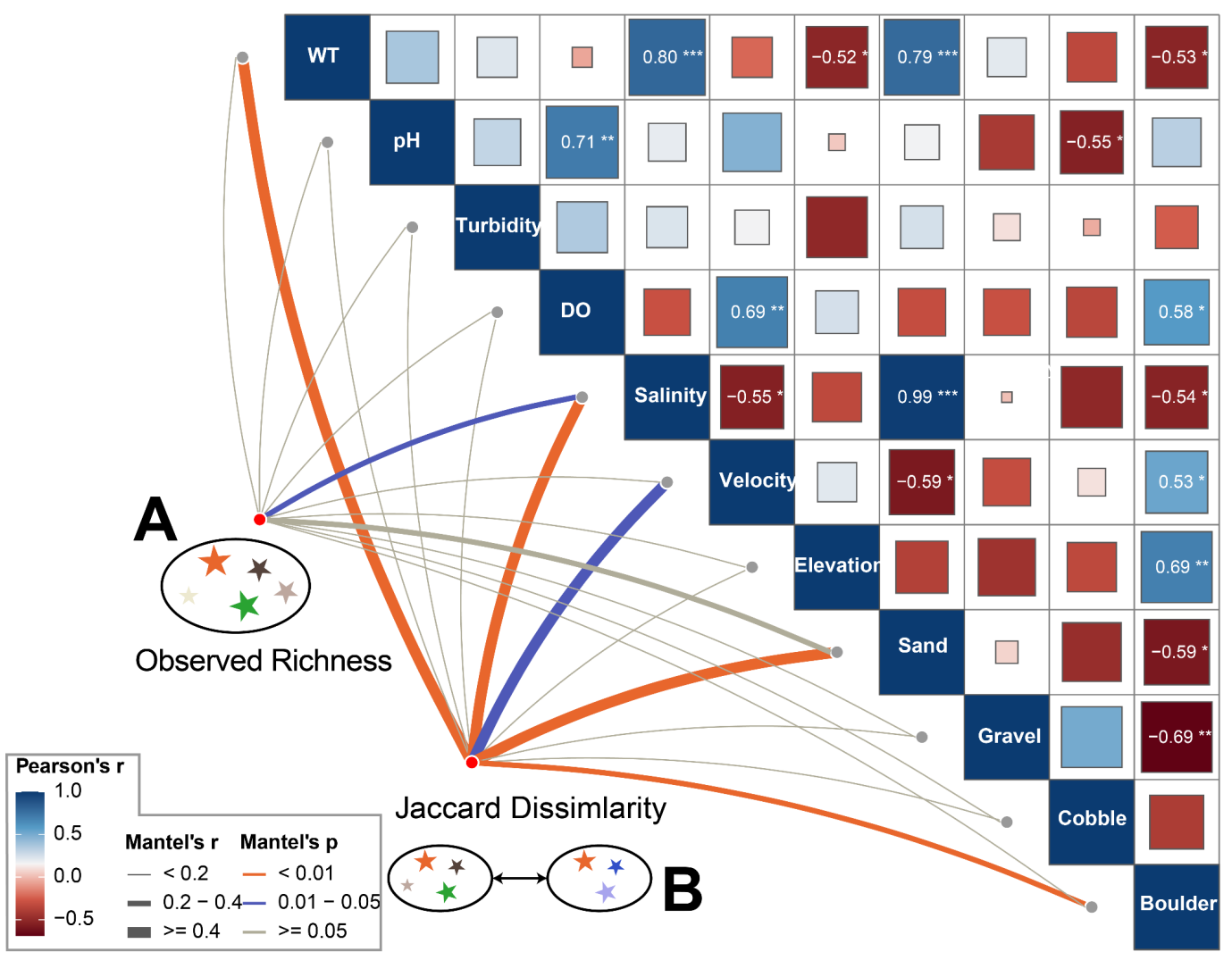


**Figure S1.** Partial Mantel tests assessing the relationship between local environmental variables and (A) species richness, and (B) community composition dissimilarity (Jaccard distance), after controlling for geographic distance. Line widths correspond to the strength of the correlation (Mantel’s r), while line colors indicate statistical significance (Mantel’s p-value).


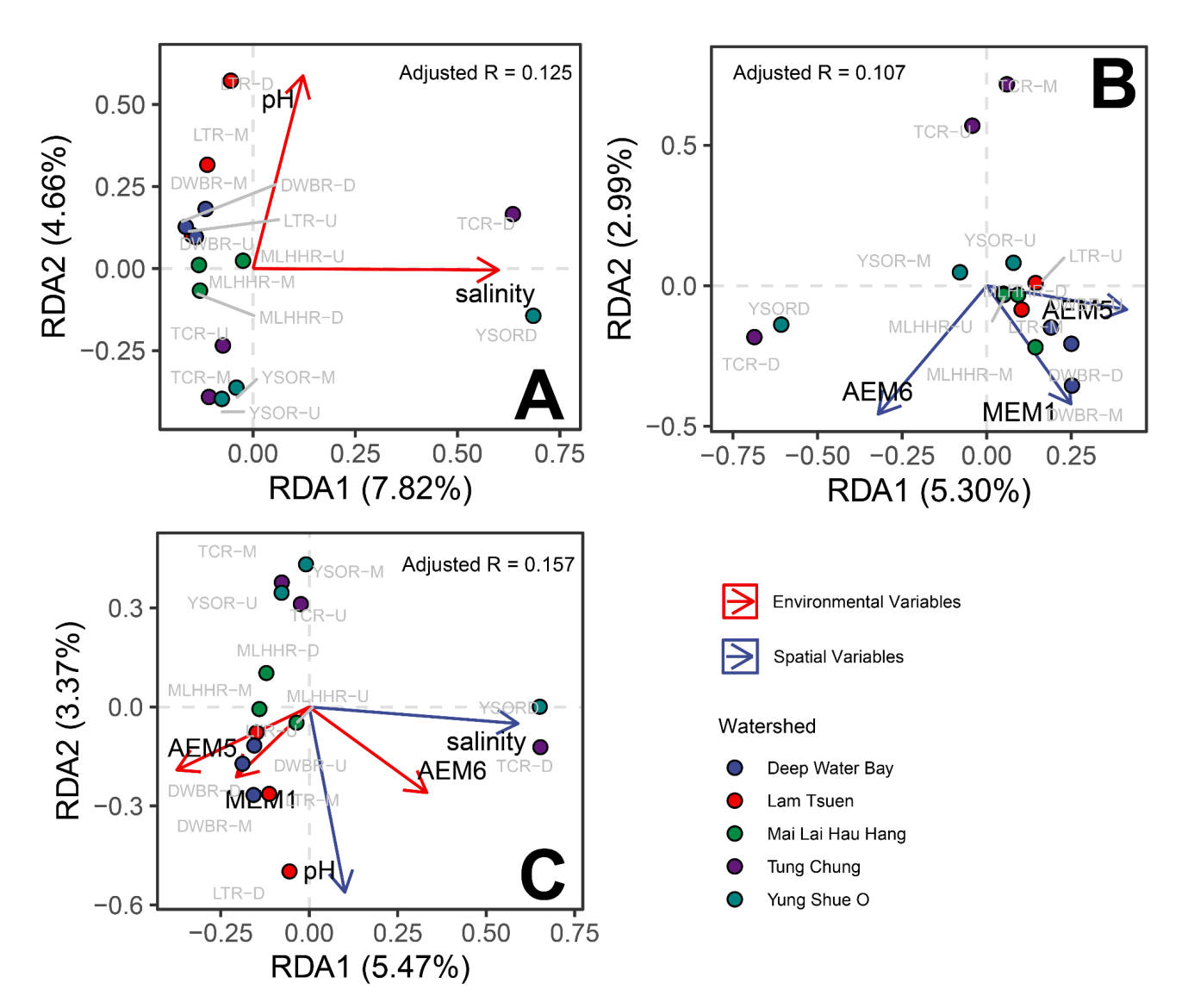


**Figure S2.** Biplots of db-RDA analysis. A. dbRDA of environmental variables by separate forward selection. B. dbRDA of spatial variables by separate forward selection. C. dbRDA of environmental and spatial variables (combined the result of separate selection).


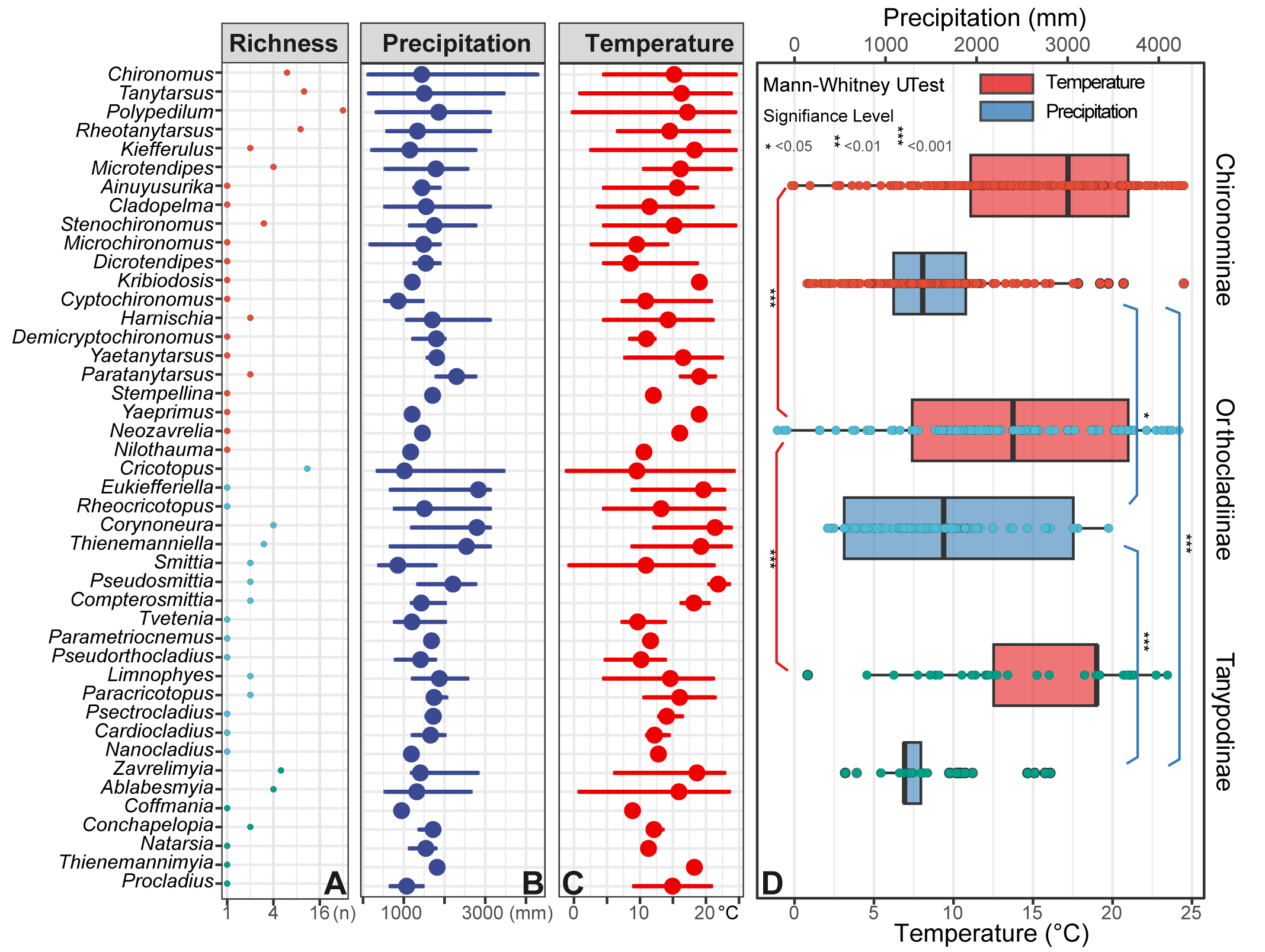


**Figure S3.**Overview of the climatic niche of chironomid species matched to Hong Kong barcodes (97% threshold), summarized at the genus and subfamily levels. From left to right: First panel: Species richness per genus. Second panel: Distribution of specimen records across annual total precipitation. Third panel: Distribution of specimen records across average minimum monthly air temperature. Fourth panel (barplot):summarized climatic niche breadth at the subfamily level. Pairwise differences in climatic variables among subfamilies were assessed using the Mann–Whitney U test.


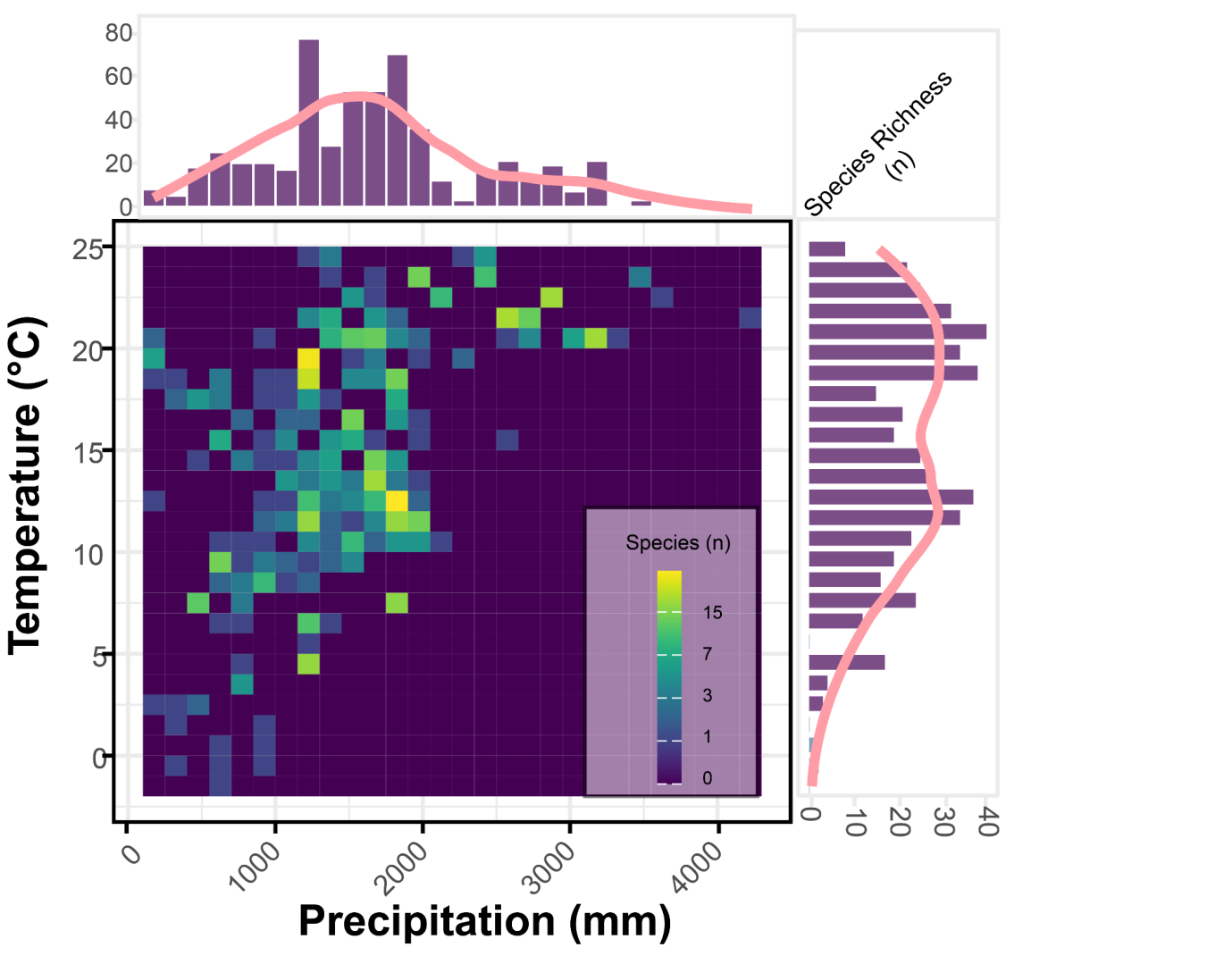


**Figure S4.**  Heat map depicting the climatic distribution of chironomid species matched with Hong Kong specimens via DNA barcoding at a 97% similarity threshold. The color intensity in the heat map reflects species richness within each climatic bin defined by annual total precipitation and average minimum monthly temperature. The top barplot and right barplot show the distribution of species richness along the gradient of precipitation and temperature, respectively. Climatic data were obtained from WorldClim at a 30 m spatial resolution for the period 2010–2019 (Fick and Hijmans, 2017)


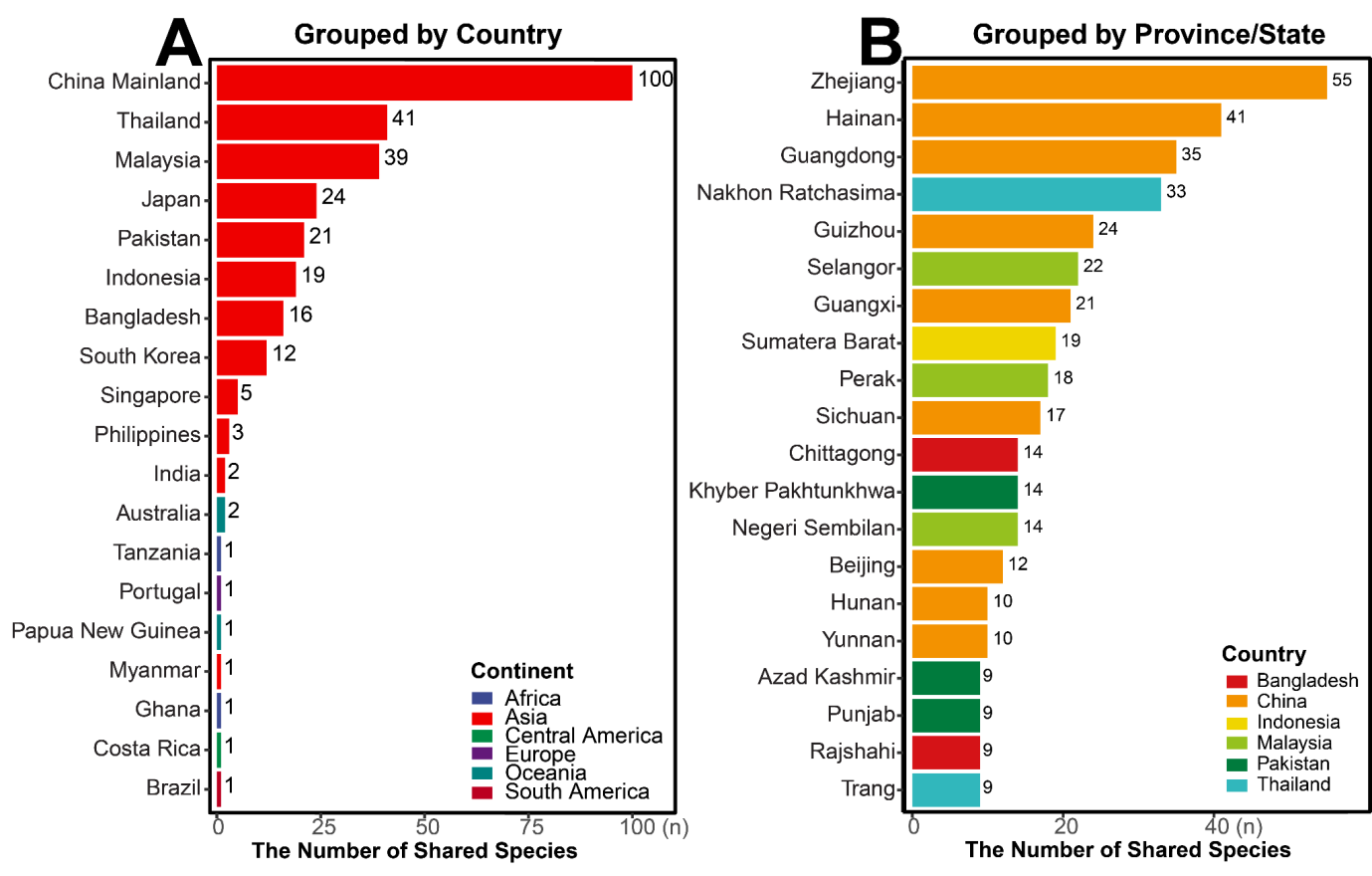


**Figure S5.** Distribution of shared species between the Hong Kong and (A) countries, and (B) subnational administrative regions (provinces or states). Data are ranked in descending order by the number of shared species; only the top 20 provinces/states are displayed.

**
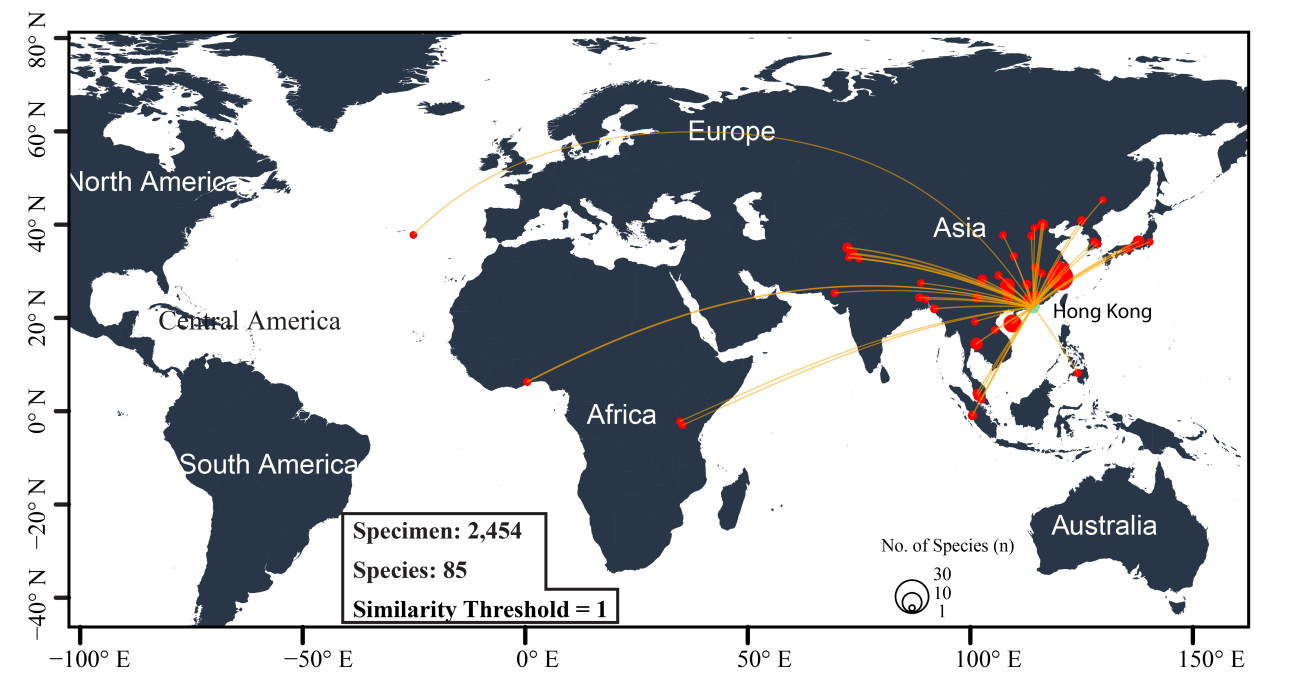
**

**Figure S6.** Cross-database barcode matching analysis between local and global DNA barcode datasets (100% identity threshold). Matched occurrence records were aggregated at the provincial or state level and connected to Hong Kong with yellow lines. The size of the red dots is proportional to the number of shared species.


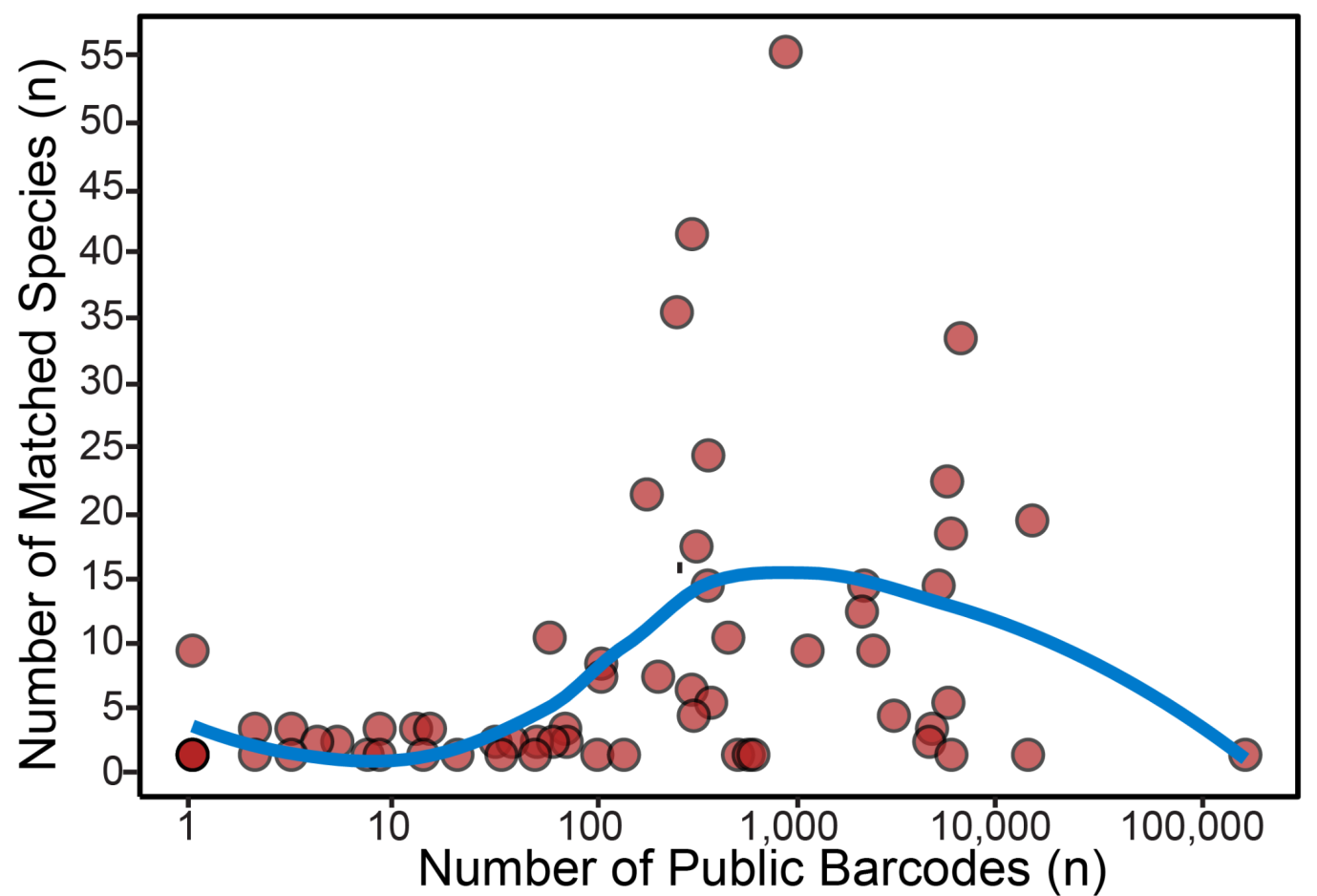


**Figure S7.** The relationship between the number of public barcode records available for each province/state and the number of species matched with those found in the Hong Kong. Specimen data were retrieved from the public library on BOLD system (as of August 2025).
